# Forging Sustainable Carbon–Nitrogen Bonds from CO_2_ and NO*_x_*: Mechanistic Insights and Catalyst Design for Electrochemical C—N Coupling

**DOI:** 10.1002/cssc.70702

**Published:** 2026-05-12

**Authors:** Soo Jin Jeon, Chengkai Xia, Jun Young Kim

**Affiliations:** ^1^ Department of Biomedical‐Chemical Engineering The Catholic University of Korea Bucheon Republic of Korea; ^2^ Department of Biotechnology The Catholic University of Korea Bucheon Republic of Korea; ^3^ School of Materials Science and Engineering Collaborative Innovation Center of Ministry of Education and Shanxi Province for High‐performance Al/Mg Alloy Materials North University of China Taiyuan China; ^4^ Department of Artificial Engineering The Catholic University of Korea Bucheon Republic of Korea

**Keywords:** catalyst design, detection methods, electrocatalysis, electrochemical C–N coupling

## Abstract

Electrochemical C—N coupling between carbon sources (CO_2_, CO) and nitrogen feedstocks (NO_*x*_) offers a sustainable route to synthesize value‐added organonitrogen compounds under ambient conditions. This strategy circumvents the high‐temperature and multistep limitations of thermochemical processes while simultaneously mitigating carbon and nitrogen pollution. Despite recent advances, the reaction suffers from slow kinetics, competing side reactions, and limited selectivity. This review highlights mechanistic insights and catalyst design principles that govern efficient C—N bond formation. We discuss reaction pathways from CO_2_/CO and NO_*x*_ precursors, identifying key intermediates (^*^COOH, ^*^CO, ^*^NO, ^*^NH_2_) through operando spectroscopy and theory. Catalyst architectures, including dual‐atom sites, heterostructure interfaces, vacancy‐engineered lattices, and single‐atom frameworks, modulate local charge distribution to favor selective coupling. At the system level, advances in reactor configuration and electrolyte optimization further enhance efficiency and stability. Finally, emerging directions in multimodal diagnostics, machine learning‐driven catalyst discovery, and technoeconomic evaluation outline a roadmap for scalable electrochemical C—N coupling. Together, these efforts establish a foundation for sustainable nitrogen chemistry and renewable‐powered carbon utilization.

## Graphical abstract

Electrochemical C—N coupling enables the direct conversion of CO_2_/CO and NO_*x*_ into value‐added organonitrogen compounds under mild and sustainable conditions. Catalyst architecture governs the coactivation of carbon and nitrogen intermediates and their interfacial coupling, dictating reaction pathways, selectivity, and efficiency. Understanding these structure–mechanism relationships is essential for designing scalable and practical electrosynthesis systems.

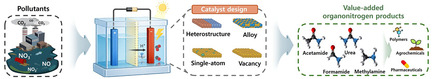

## 1 Introduction

Since the onset of the industrial revolution, relentless fossil fuel combustion has driven atmospheric CO_2_ and CO concentrations to record levels, intensifying global warming, sea‐level rise, and extreme weather phenomena [1, 2]. Concurrently, industrial NO_*x*_ emissions and agricultural NO_3_^−^ runoff have polluted waterways, spurred eutrophication, and acidified soils [3]. Transforming these carbon‐ and nitrogen‐based pollutants into valuable organonitrogen products, i.e., ranging from fertilizers and pharmaceuticals to specialty polymers, not only mitigates environmental damage but also creates economic value [4, 5].

Electrochemical C—N coupling emerges as a promising one‐step approach under ambient conditions, in stark contrast to traditional high‐temperature, high‐pressure, or hazardous‐reagent syntheses [6]. Unlike conventional thermochemical processes that operate at high temperature, high pressure, or with hazardous reagents, electrochemical approaches exploit renewable electricity to drive bond formation at ambient conditions [7]. This methodology not only minimizes energy consumption and CO_2_ footprint but also enables process modularity and on‐demand operation [8, 9]. However, achieving efficient C—N bond formation remains challenging. The inert N≡N bond in molecular nitrogen limits its activation, while parasitic hydrogen evolution competes for electrons and protons [10, 11]. Consequently, attention has shifted toward more reactive nitrogen oxides (NO_3_^−^, NO_2_^−^, NO), which reduce at lower overpotentials and are readily available from wastewater streams [12, 13]. Their electrochemical valorization simultaneously mitigates nitrate pollution and produces valuable nitrogen‐containing organics, delivering a dual environmental benefit.

At the heart of this chemistry lies the rational design of electrocatalysts that can coactivate carbon and nitrogen precursors in a synchronized manner. The ideal catalyst must selectively adsorb and activate both species, orchestrate multielectron transfer, and suppress competing pathways such as CO_2_‐to‐CO or NO_*x*_‐to‐NH_3_ reduction [14]. Recent advances in electronic‐structure engineering have demonstrated that catalyst architecture critically dictates the reaction landscape. Heterostructure engineering enhances interfacial charge transfer and tailors intermediate adsorption [15, 16]; alloying modulates the d‐band center to tune binding strength [17, 18]; vacancy creation generates coordinatively unsaturated sites that facilitate ^*^CO and ^*^NH_2_ stabilization [19, 20]; and single‐atom catalysts maximize atom utilization while offering precise geometric control [21, 22]. These strategies collectively mitigate competing hydrogen evolution by modulating the local electric field and optimizing reactant coverage. Furthermore, the electrolyte composition, pH, and applied potential serve as external knobs to steer selectivity toward C—N coupling rather than parallel reduction reactions [23].

Beyond purely electrochemical systems, photoelectrochemical (PEC) platforms have broadened this paradigm by coupling light absorption with catalytic redox chemistry. Under solar illumination, semiconducting photoelectrodes generate photogenerated carriers that lower activation barriers for both carbon and nitrogen reduction, improving energy efficiency and selectivity [24, 25]. Tailoring the atomic composition and band alignment of photoelectrodes through doping, heterojunction formation, and surface reconstruction enhances charge separation and facilitates C—N bond formation under bias‐free or bias‐assisted operation. For instance, integrating plasmonic nanostructures or cocatalyst overlayers can extend light absorption into the visible region and enhance photocatalytic activity. Although still at an early stage, PEC‐driven C—N coupling highlights a promising route toward solar‐to‐chemical conversion of CO_2_ and NO_*x*_, fully powered by renewable photons [26]. Nevertheless, to maintain conceptual clarity, this review treats PEC systems as an extension of electrochemical principles rather than a separate mechanistic category, emphasizing that both rely on the same fundamental sequence of adsorption, proton‐coupled electron transfer (PCET), and C—N coupling at the solid–liquid interface.

Recent mechanistic insights have begun to clarify the elementary steps governing electrochemical C–N coupling. Operando infrared, Raman, and X‐ray absorption spectroscopy have captured transient intermediates (^*^COOH, ^*^CO, ^*^NOH, ^*^NH_2_) and revealed potential‐dependent coupling sequences [27]. The combination of density‐functional‐theory calculations and kinetic modeling further quantifies the energy landscape of each pathway, identifying ^*^CO + ^*^NH_2_ → ^*^CONH as a frequently rate‐determining step [28]. These findings underpin data‐driven catalyst discovery, enabling predictive design guided by electronic descriptors such as adsorption energy, charge‐transfer resistance, and work‐function alignment.

Herein, we provide a comprehensive overview of recent advances in electrochemical C—N coupling for the sustainable synthesis of value‐added organonitrogen compounds from waste‐derived carbon and nitrogen sources. First, we elucidate the reaction mechanisms underpinning organonitrogen synthesis from diverse carbon (CO_2_, CO) and nitrogen (NO_3_^−^, NO_2_^−^, NO) feedstocks. Next, we survey experimental observation methods, including in situ spectroscopy and high‐performance liquid chromatography (HPLC), for the precise detection and quantification of C—N coupled products. Subsequently, we compile and critically assess recently published synthetic approaches aimed at maximizing C—N product yields through advanced catalysts. Finally, we present our outlook, highlighting challenges and opportunities in organonitrogen production, and explore how this utilization can facilitate the integration of renewables into key sectors, including energy conversion and environmental sustainability. Unlike previous reviews that mainly summarize catalyst classes or product trends, this review bridges three dimensions of electrochemical C—N coupling that are often treated independently: (i) mechanistic pathways associated with distinct C—N products, (ii) the capabilities and limitations of analytical techniques for identifying intermediates and products, and (iii) catalyst and reaction‐engineering strategies that govern intermediate generation and coupling. Rather than simply describing these aspects in isolation, this work explicitly links reaction mechanisms with experimental observables and catalyst design principles, thereby enabling a more consistent interpretation of reported results. In this way, we establish an integrated framework that not only rationalizes existing studies but also provides guidance for the design of selective and efficient electrochemical C—N coupling systems.

## 2 Reaction Mechanisms on Organonitrogen Compound Production

**FIGURE 1:**
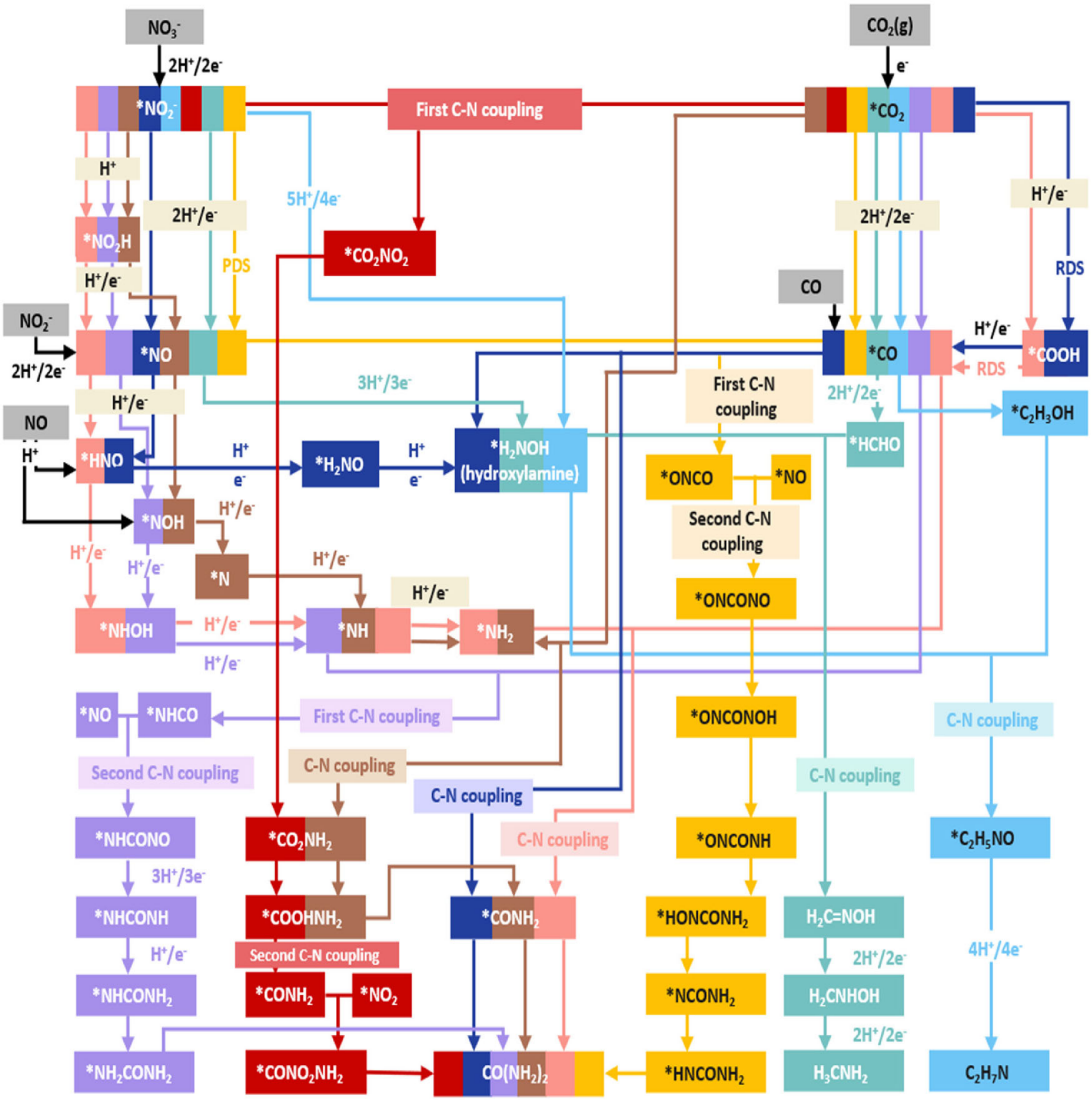
Schematic of electrochemical C—N coupling pathways with carbon (CO_2_/CO) and nitrogen (NO_*x*_) feedstocks. Adapted with permission from Yu et al. [29]. Copyright 2025, Elsevier.

Electrochemical formation of C—N bonds provides the molecular foundation enabling sustainable organic nitrogen synthesis. In this process, carbon sources (CO, CO_2_) and nitrogen sources (NO, NO_2_^–^, NO_3_^–^) are simultaneously adsorbed onto the electrode surface, followed by the formation of reactive intermediates such as ^*^CO, ^*^COOH, and ^*^NH_2_ through stepwise PCET reactions [29]. These adsorbed species interact at the electrode interface via heteroatoms, resulting in the formation of C–N bonds that lead to various organic nitrogen compounds such as urea, formamide, and acetamide. Figure 1 illustrates the schematic structure of this overall reaction mechanism. The thermodynamic and kinetic characteristics of each reaction step depend significantly not only on the chemical properties of the carbon and nitrogen sources but also on the local coordination environment and the interfacial electric field. Therefore, understanding how these variables influence the coupling steps remains a key challenge for designing high‐performance catalysts.

### 2.1 Electrochemical Preconversion of Carbon and Nitrogen Feedstocks

The pretransition step refers to the electrochemical activation of carbon‐ and nitrogen‐containing reactants, during which they are converted into partially reduced intermediates adsorbed on the catalyst surface [6]. This step is essential for C—N bond formation because fully oxidized species or already sufficiently reduced products generally exhibit insufficient reactivity to form heteroatomic bonds under electrochemical conditions [30].

Under cathodic polarization, CO_2_ reduction proceeds through an adsorbed carboxyl intermediate (^*^COOH) that undergoes PCET to yield ^*^CO, with a typical free‐energy barrier of 0.40–0.50 e [31, 32]. The resulting ^*^CO can either desorb as carbon monoxide or participate in further PCET steps to form partially hydrogenated species such as ^*^CHO, ^*^CH_2_O, or ^*^CH_3_O [29]. These intermediates act as electrophilic anchors for nucleophilic attack by nitrogen‐derived species, establishing the first C—N bond.

Although CO and CO_2_ share similar reduction intermediates, their activation mechanisms differ subtly. CO, lacking the initial carboxyl formation step, can adsorb directly via π‐backbonding to metal centers, resulting in distinct surface coverage and reaction kinetics [33]. Treating CO and CO_2_ as interchangeable carbon sources simplifies mechanistic modeling, but precise potential‐dependent behavior must be considered to predict product distribution accurately. Advanced kinetic simulations now demonstrate that under dynamic electrolysis conditions, transient accumulation of ^*^CO intermediates can strongly influence the probability of C—N bond formation events [34].

Reactive nitrogen oxides (NO_*x*_) offer substantially lower activation barriers than molecular N_2_ due to weaker N—O bonds and higher aqueous solubility. While NO_2_^−^‐fed systems often exhibit faster kinetics owing to fewer electron‐transfer steps, NO_3_^−^ remains the more abundant and environmentally relevant substrate. Its widespread presence in wastewater provides an opportunity for integrated pollutant valorization, converting harmful nitrates into industrially useful nitrogen‐containing chemicals. However, nitrate reduction frequently suffers from competing hydrogen evolution and incomplete conversion to ^*^NH_2_ species [35]. Nitrate (NO_3_^−^) reduction typically follows a multistep PCET cascade:




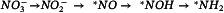




Each step involves progressive protonation and electron transfer with cumulative free‐energy barriers of 0.30–0.45 eV [36]. Among these intermediates, ^*^NOH and ^*^NH_2_ are widely recognized as the most reactive toward carbon species. Density‐functional‐theory (DFT) calculations reveal that the elementary coupling ^*^CO + ^*^NOH → ^*^CONOH requires only 0.12 eV, followed by protonation to ^*^CONH_2_ (ΔG‡ ≈ 0.07 eV), underscoring the kinetic accessibility of dual C–N bond formation [37].

### 2.2 Electrochemical C—N Coupling Pathways

Electrochemical C—N coupling pathways describe the sequence by which partially reduced carbon‐ and nitrogen‐derived intermediates converge at the electrode interface to form heteroatomic bonds. Rather than direct coupling between fully oxidized or fully hydrogenated species, effective C—N bond formation typically occurs after both reactants undergo pre‐conversion to surface‐bound intermediates with complementary reactivity [38].

Computational studies and isotope‐labeling experiments consistently identify the coupling of ^*^CO and ^*^NH_2_ to form ^*^CONH as the rate‐determining step (ΔG‡ ≈ 0.50 eV) [39]. This pathway aligns with the Sabatier principle, wherein an optimal adsorption strength ensures sufficient stabilization of intermediates without impeding turnover [40]. For example, Cu‐based surfaces with moderate d‐band occupancy favor ^*^CO binding while simultaneously facilitating ^*^NH_2_ activation, whereas more oxophilic metals such as Bi or Sn stabilize ^*^COOH and ^*^HCOO, altering the balance between reductive hydrogenation and coupling [41]. The thermodynamic and kinetic characteristics of key C—N coupling steps have been quantitatively analyzed based on representative theoretical studies. For the commonly proposed ^*^CO + ^*^NH_2_ → ^*^CONH_2_ pathway, the activation energy barrier has been reported to span a wide range depending on catalyst composition and active site structure. Specifically, relatively low barriers of around 0.2–0.4 eV have been obtained on dual‐site or heterostructured catalysts (e.g., Fe–Ni diatomic sites or Cu—Ni interfaces) [42, 43], whereas significantly higher barriers (>1.0 eV) are observed on monometallic surfaces such as pure Cu or Ni [43]. In contrast, alternative coupling pathways involving oxygenated nitrogen intermediates, such as ^*^CO + ^*^NOH → ^*^CONOH, typically exhibit higher activation barriers [37]. For example, a barrier of around 0.97 eV has been reported on Cu/Cu_2_O heterostructures, suggesting comparatively sluggish kinetics for this route [44]. This trend is further supported by thermodynamic analysis: the ^*^CO + ^*^NH_2_ coupling step is often exergonic (e.g., Δ*G* ≈ –0.24 eV on Ru_1_Cu systems), whereas the ^*^CO + ^*^NOH pathway is generally endergonic (e.g., Δ*G* ≈ + 0.62 eV), indicating less favorable energetics [45]. These differences can be attributed to the stronger adsorption and higher surface coverage of ^*^NH_2_ under reducing conditions, compared to the relatively unstable and transient nature of ^*^NOH species. As a result, ^*^NH_2_ is more readily available for coupling with ^*^CO, leading to both lower activation barriers and more favorable reaction energetics. Furthermore, catalyst‐dependent factors, including electronic structure, coordination environment, and interfacial effects, play a critical role in modulating these energetics. For instance, bifunctional or dual‐site catalysts can spatially separate ^*^CO and ^*^NH_2_ adsorption, thereby significantly lowering the coupling barrier [43]. Overall, these quantitative comparisons consistently indicate that the ^*^CO + ^*^NH_2_ pathway is both kinetically and thermodynamically more favorable than ^*^CO + ^*^NOH across a wide range of catalyst systems, although the absolute energy values remain highly sensitive to catalyst structure, surface facet, and solvation effects.

In practice, distinct reaction pathways emerge depending on the target product, governed by the formation, stability, and interfacial interactions of key carbon‐ and nitrogen‐derived intermediates. To provide a unified understanding, C—N coupling reactions can be systematically classified into distinct pathway types based on the formation, transformation, and interaction of key intermediates. Within this framework, three representative pathway types can be identified, corresponding to urea‐, amide‐, and amine‐forming reactions.

For urea synthesis, the CO_2_ reduction reaction (CO_2_RR) typically proceeds via a carbon pathway involving ^*^COOH → ^*^CO [46], while nitrate/nitrite reduction follows a stepwise nitrogen pathway of ^*^NO_3_ → ^*^NO_2_ → ^*^NO → ^*^NH_2_ [47]. In this context, ^*^CO serves as a relatively stable carbon intermediate that can accumulate at the interface, whereas ^*^NO_2_ and ^*^NO act as reactive nitrogen species capable of undergoing further reduction or participating directly in C—N coupling. The coexistence and spatial proximity of these intermediates critically determine not only the probability of C—N bond formation but also the competition with hydrogenation pathways (e.g., ^*^CO → ^*^CHO and ^*^NO_2_ → ^*^NH_2_). In particular, coupling between ^*^CO and ^*^NO_2_ can yield adsorbed intermediates such as ^*^CONO_2_ or ^*^CO–^*^NO_2_, which are subsequently hydrogenated to ^*^CONH_2_ or NHCONH species before forming urea [48]. Both the initial ^*^CO–^*^NO_2_ coupling step and the subsequent hydrogenation of the coupled intermediate involve relatively high energy barriers, with the coupling step often identified as the rate‐determining step. Accordingly, the surface coverage and adsorption strength of ^*^CO and ^*^NO_2_ emerge as key kinetic descriptors. In addition, alternative branching pathways involving CONH‐centered intermediates, followed by further coupling with NH_2_ species, have been proposed, where the stabilization of these intermediates plays a decisive role in product selectivity.

For amide formation, two representative pathways can be distinguished. In the formamide‐type pathway, ^*^CO generated from CO_2_RR undergoes further hydrogenation to carbonyl intermediates such as ^*^CHO and ^*^CH_2_O, which subsequently couple with ^*^NH_2_ to form ^*^CONH_2_, eventually yielding formamide [6]. The ^*^CO → ^*^CHO transformation typically proceeds via PCET, and the subsequent evolution of ^*^CONH_2_ involves either further hydrogenation or desorption. In many cases, the hydrogenation and stabilization of ^*^CONH_2_ or CONH_*x*_ intermediates present higher energetic barriers than the initial C—N bond formation, thereby acting as the rate‐limiting step [49]. In contrast, the acetamide‐type pathway involves the formation of C_2_ intermediates, where ^*^CO is reduced to ^*^CHO and further to acetaldehyde (CH_3_CHO), which can desorb into the solution phase [50]. The dissolved acetaldehyde then reacts with NH_2_OH, produced via nitrate reduction, to form acetaldoxime (CH_3_CH=NOH). This oxime intermediate subsequently undergoes dehydration to acetonitrile (CH_3_CN), followed by hydrolysis to yield acetamide. In this route, either the dehydration of the oxime or the hydrolysis of the nitrile often constitutes the rate‐limiting step. Notably, local pH, interfacial electric fields, and the accumulation of reactive species strongly influence the activation barriers associated with these transformations.

For amine production, a distinct mechanism is governed by oxygenated carbon intermediates and partially reduced nitrogen species. In this pathway, the key intermediates are formaldehyde (HCHO) and ^*^NH_2_OH, rather than ^*^CO or ^*^NH_2_. Specifically, ^*^CO produced from CO_2_RR can be further hydrogenated to form HCHO, while nitrate reduction proceeds through ^*^NO_2_ and ^*^NO intermediates to generate NH_2_OH [29]. These two species can then react near the interface through nucleophilic condensation to form formaldoxime, which acts as the central C—N coupled intermediate. Unlike direct surface‐mediated coupling pathways, this mechanism is largely dominated by a solution–interface reaction between HCHO and NH_2_OH, followed by a stepwise hydrogenation sequence [51]. Notably, the initial C—N bond formation is typically spontaneous, whereas the subsequent hydrogenation of oxime intermediates is more likely to govern the overall reaction rate.

Collectively, the product‐dependent mechanisms differ not only in the identity of the key C‐ and N‐containing intermediates, but also in the step that most strongly limits the overall rate. Urea formation is generally governed by the surface coadsorption and coupling of ^*^CO‐derived and NO_*x*_‐derived intermediates [44], whereas formamide formation more often depends on the subsequent hydrogenation/stabilization of carbonyl–nitrogen coupled species [52]. In contrast, amine formation is frequently mediated by solution/interfacial condensation of oxygenated carbon species with partially reduced nitrogen intermediates, followed by hydrogenation steps [53]. Thus, the dominant kinetic bottleneck shifts from interfacial coupling to postcoupling conversion depending on the target product class. If carbon and nitrogen intermediates are generated at mismatched rates or reside on spatially segregated domains, the probability of hetero‐coupling decreases dramatically. Dual‐functional catalysts address this limitation by integrating carbon‐ and nitrogen‐activation sites, thereby synergistically lowering kinetic barriers. For example, a CuO_*x*_/BiO_*x*_ nanocomposite has been reported to provide spatially distinct yet electronically coupled active sites, where BiO_*x*_ domains preferentially facilitate CO_2_ adsorption and reduction toward ^*^CHO intermediates via the ^*^HCOO pathway, while adjacent CuO_*x*_ sites promote the stepwise hydrogenation of NO_*x*_^–^ to ^*^NH_2_ species. The nanocomposite architecture ensures nanoscale proximity between these active sites, shortening the diffusion distance of reactive intermediates and enabling sequential intermediate generation and coupling [27]. In addition, DFT calculations indicate that the presence of dual active sites reduces the thermodynamic barriers associated with ^*^CHO and ^*^NH_2_ formation while favoring their coupling over competing hydrogenation pathways [54]. As a result, the probability of ^*^CHO–^*^NH_2_ coupling is significantly enhanced, leading to improved C—N bond formation efficiency.

Similarly, bimetallic systems, particularly Cu—Ni‐based catalysts, have been reported to exhibit spatially separated yet electronically coupled active sites, in which Cu sites stabilize carbon‐derived intermediates (e.g., ^*^CO) generated during CO_2_ reduction, while adjacent Ni sites facilitate the activation and stepwise hydrogenation of nitrogen species [43]. Such a configuration ensures nanoscale proximity between the active centers, thereby shortening the diffusion distance of reactive intermediates and promoting their sequential generation and interfacial coupling. In addition, a conductive and chemically interactive local environment further stabilizes key intermediates and modulates the interfacial electronic structure, enhancing intermediate adsorption while suppressing competing side reactions such as hydrogen evolution [43, 55].

Collectively, these findings highlight that spatially separated yet electronically coupled active sites represent a broadly applicable design strategy for promoting efficient C—N coupling.

Electrochemical C—N coupling mechanisms have advanced considerably, yet disparate interpretations are still reported due to variations in catalyst structure, electrolyte composition, and experimental conditions. To consistently understand these differences, a unified perspective that considers three complementary levels is required. 1) elementary step thermodynamics – quantitative adsorption energies and activation barriers for ^*^CO_*x*_ and ^*^NH_*x*_ species, 2) interfacial charge dynamics – potential‐dependent electron transfer and electric‐double‐layer modulation influencing PCET rates, and 3) macroscopic transport effects – diffusion and convection determining local reactant concentrations and pH gradients.

In summary, the electrochemical formation of C—N bonds proceeds through a finely orchestrated sequence of adsorption, activation, and coupling events that depend sensitively on both catalyst structure and reaction environment. Emerging evidence from theory and operando studies reveals that the interplay between carbon and nitrogen activation pathways can be tuned by engineering surface coordination, interface polarity, and local electric fields. Integrating these mechanistic insights into catalyst and system design will accelerate the development of next‐generation electrochemical platforms capable of producing organonitrogen compounds with high selectivity, productivity, and sustainability.

### 2.3 Mechanism of Photoelectrochemical C—N Coupling

**FIGURE 2:**
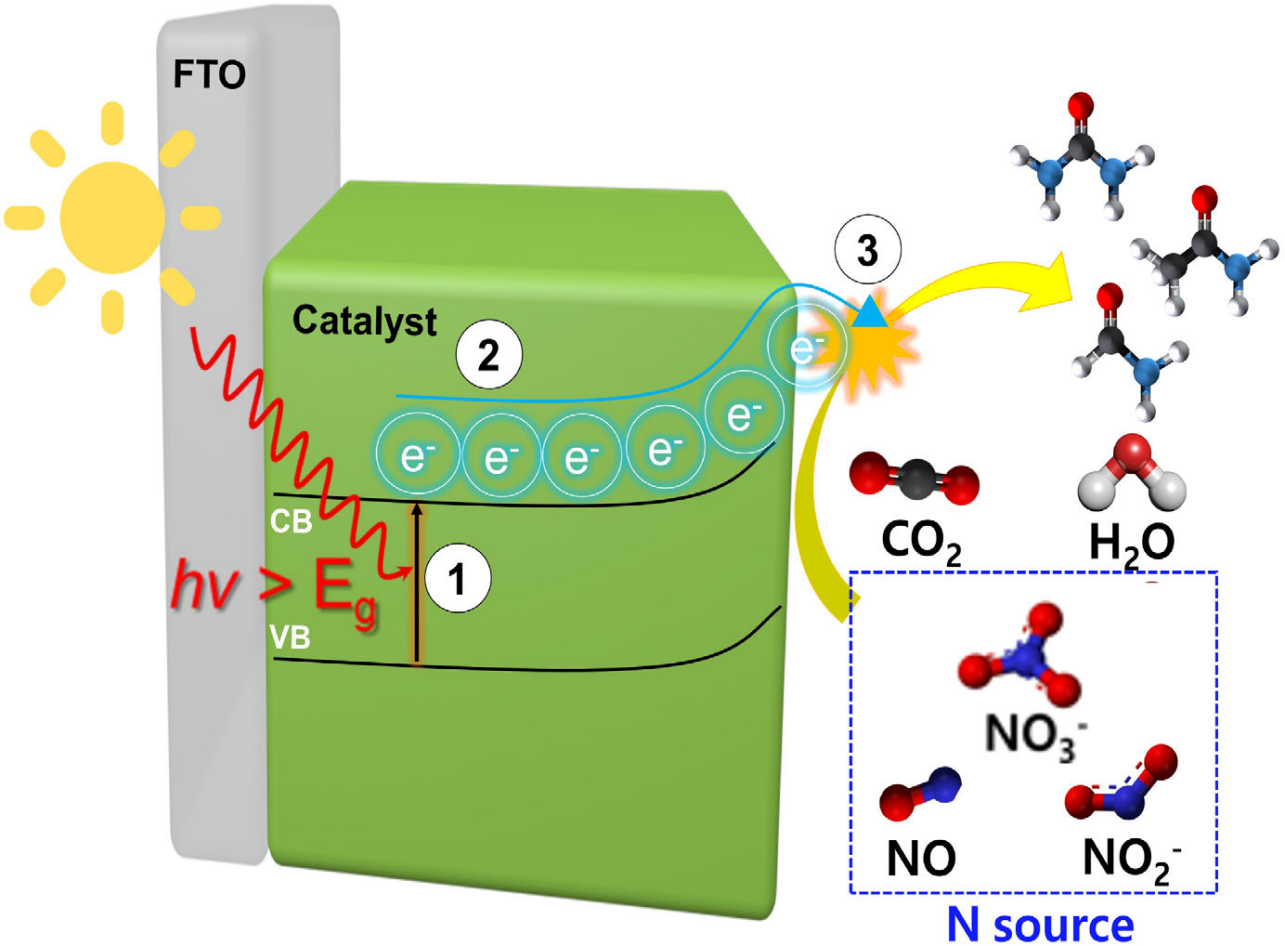
Representative example of PEC C—N coupling over a SAC Cu‐TiO_2_ photocathode. Schematic illustration of the PEC process. Adapted with permission from Han et al. [56]. Copyright 2025, Springer Nature.

Photoelectrochemical (PEC) C—N coupling has emerged as an appealing extension of conventional electrocatalytic systems by integrating light harvesting with interfacial redox chemistry. The photoelectrochemical reaction begins with light absorption by the semiconductor, generating electron–hole pairs when the photon energy exceeds the band gap. The photogenerated electrons are then separated under an applied bias and transported to the catalytic sites, where the coreduction of CO_2_ and nitrogen sources leads to the formation of C—N coupling products (Figure 2). Efficient charge separation and electron transport, while suppressing electron–hole recombination, are critical for achieving high PEC performance [56]. More specifically, the PEC process can be divided into three fundamental steps: (i) light absorption and the generation of electron‐hole pairs, (ii) charge separation and migration toward the semiconductor‐electrolyte interface, and (iii) surface redox reactions driven by the accumulated charge carriers. These processes are strongly governed by the charge transport properties of the semiconductor and the interfacial energy alignment [57]. Although PEC systems involve additional photophysical steps, their interfacial C—N coupling mechanisms share key similarities with electrochemical systems. Accordingly, this section highlights PEC‐specific aspects, while the main discussion of this review remains focused on electrochemical C—N coupling.

In contrast to purely electrochemical processes, PEC systems introduce an additional photophysical dimension, in which light absorption, charge separation/transport, and surface coupling reactions collectively determine overall performance. This feature offers the possibility of lowering the external energy input while simultaneously improving the directionality of charge flow toward the catalytic interface. However, PEC C—N coupling remains at an early stage of development, and its practical implementation is still constrained by limited light utilization, rapid electron–hole recombination, modest current density, and the persistent trade‐off between activity and long‐term stability [58]. For example, Zheng et al. developed a photoelectrochemical system by introducing atomically dispersed Cu into TiO_2_, thereby constructing closely coupled Cu/Ti dual active sites that enable efficient urea synthesis from CO_2_ and nitrate [59]. In this system, Cu sites selectively generate ^*^CO intermediates, while adjacent Ti sites facilitate the reduction of nitrate to ^*^NH_2_ species, promoting interfacial C—N coupling between these intermediates. Under illumination with an applied bias of −0.5 V, the system achieved a Faradaic efficiency of 68% and a urea formation rate of 52.45 μmol L^−1^ h^−1^, significantly outperforming the corresponding purely electrochemical system. *In situ* Fourier‐transform infrared (FTIR) and DFT analyses further revealed that the ^*^CO–^*^NH_2_ coupling pathway exhibits a lower energy barrier than competing hydrogenation reactions, which is attributed to the nanoscale proximity and electronic interaction of the dual active sites. This study highlights how PEC‐specific charge generation and spatially coupled active sites can synergistically enhance intermediate availability and coupling probability, thereby improving C—N coupling efficiency.

In this context, improving charge carrier separation and transport while suppressing electron–hole recombination remains a critical challenge, and further development of strategies such as heterojunction design, cocatalyst integration, and material optimization is essential for achieving high efficiency and improved reaction performance in PEC C–N coupling systems.

## 3 C—N Coupled Products Detection Methodologies

Generally, the comprehensive analysis of reaction intermediates and products is essential for understanding reaction mechanisms and evaluating the performance of electrochemical C—N coupling systems. A typical detection strategy for electrochemical C—N coupled products employs operando vibrational spectroscopy (ATR‐SEIRAS, in‐situ Raman), colorimetric ultraviolet‐visible (UV–vis) assays (DAMO, indophenol blue), chromatographic separations (HPLC, GC–MS), and complementary structural techniques (NMR). These methods allow reliable identification and quantification of intermediates and products while maintaining applicability to operando conditions [15, 45, 60, 61]. Table 1 summarizes representative detection methodologies, along with their strengths and limitations, including limits of detection/quantification (LOD/LOQ).

**TABLE 1:** Representative methodologies for detecting C–N coupled products.

Methodologies	Strengths	Limitations	LOD/LOQ
ATS‐SEIRAS [15]	Real‐time monitoring, surface sensitivity	Requires IR‐transparent electrodes	˜10^−6^ M
In situ Raman [60]	Molecular fingerprints, operando capability	Fluorescence background interference	˜10^−5^ M
UV–vis (DAMO) [61]	Colorimetric quantification	Interference from metal cations	˜5 μM
UV–vis (Indophenol) [19]	High selectivity for NH_3_	Enzymatic variability	˜1 μM
HPLC [62]	Multianalyte separation and detection	Complex sample preparation	˜1–10 μM
GC–MS [63]	High selectivity for volatiles	May require derivatization	˜1 ng on column
DEMS [48]	Real‐time detection of volatile species	Limited to volatile species, low signal‐to‐noise ratio	˜5 μM
NMR (^1^H/^13^C/^15^N) [45]	Unambiguous structural validation	Low sensitivity and long acquisition time	˜100 μM

### 3.1 In Situ Spectroscopy

In situ spectroscopic techniques are widely used to investigate electrochemical C—N bond formation, as they enable direct observation of reaction intermediates under operating conditions [64]. These approaches make it possible to monitor the formation and transformation of active surface species in real time, as well as their potential‐dependent behavior, which helps to address the limitations of ex situ analysis [65].

Among various in situ techniques, attenuated total reflectance surface‐enhanced infrared absorption spectroscopy (ATR‐SEIRAS) and Raman spectroscopy are widely employed to investigate interfacial reaction intermediates at electrode–electrolyte interfaces [66]. ATR‐SEIRAS offers high sensitivity to vibrational modes of surface‐adsorbed species and detects subtle changes in the electronic environment of metal active sites [67]. In contrast, Raman spectroscopy provides complementary information, revealing symmetry alterations and the emergence of new vibrational features associated with C—N bond formation [68].

**FIGURE 3:**
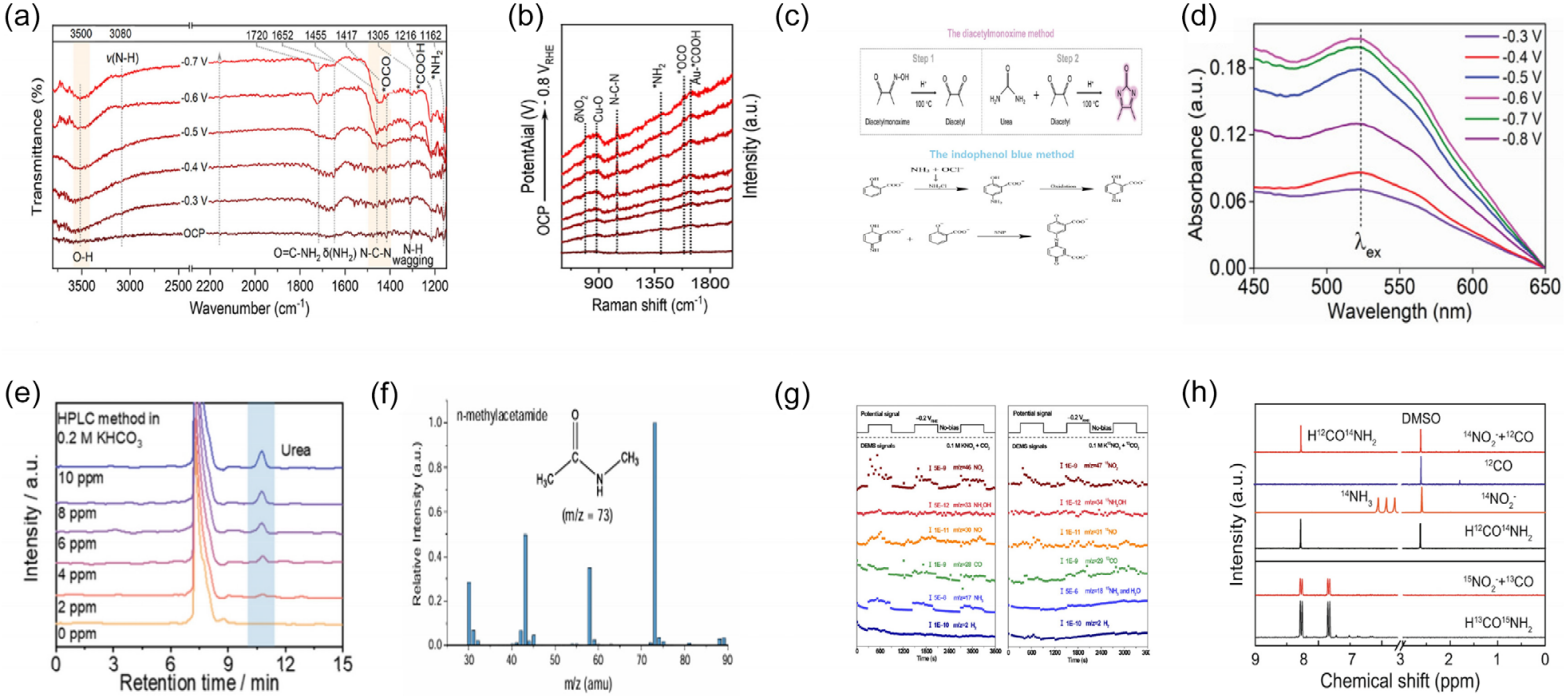
Representative analytical techniques for detecting C—N coupled products in electrochemical systems. (a, b) In situ FTIR and Raman analyses track the evolution of surface‐bound C—N intermediates under different potentials. Adapted with permission from Zhao et al. [15]. Copyright 2025, ACS. (c) Schematic diagram of urea detection by the diacetyl monoxime and urease‐based colorimetric methods. Adapted with permission from Wan et al. [60]. Copyright 2025, Wiley‐VCH GmbH. Adapted with permission form Hu et al. [141]. Copyright 2025, RSC. (d) UV–vis absorption spectra used for spectrophotometric quantification of urea concentration. Adapted with permission from Mukherjee et al. [61]. Copyright 2025, Wiley. (e) HPLC profiles confirming urea generation with concentration‐dependent peak intensity. Adapted with permission from Huang et al. [62]. Copyright 2025, Elsevier. (f) GC–MS detection of *N*‐methylacetamide *via* its characteristic fragmentation pattern. Adapted with permission from Jouny et al. [63]. Copyright 2025, Springer Nature. (g) Operando DEMS analysis of CuWO_4_ under CO_2_/nitrate coreduction conditions, showing real‐time mass signals. Adapted with permission from Linic et al. [48]. Copyright 2023, Springer Nature. (h) NMR spectra validating the isotopic origin of C—N coupling products. Adapted with permission from Lan et al. [45]. Copyright 2025, Springer Nature.

A representative study on the coreduction of CO_2_ and NO_3_^−^ over an RP–CuAu catalyst employed both ATR‐SEIRAS and in‐ situ Raman spectroscopy to elucidate the reaction mechanism [15]. The ATR‐SEIRAS spectra (Figure 3a) exhibited potential‐dependent growth of characteristic vibrational bands at 1720 cm^−1^ (O=C—NH_2_) and at 1455 and 1305 cm^−1^ (N—C—N, C—N), signifying the progressive accumulation of amide‐type intermediates on the catalyst surface. In addition, in situ Raman spectra (Figure 3b) revealed signals assigned to ^*^COOH and ^*^NH_2_ intermediates, providing direct evidence of C—N bond formation.

Collectively, these in situ techniques enable real‐time monitoring of changes in C—N intermediates, highlighting the importance of catalyst design in stabilizing key species. Thus, the synergistic application of ATR‐SEIRAS and Raman spectroscopy represents a powerful strategy for elucidating reaction pathways, controlling product selectivity, and optimizing the structure of C—N coupling electrocatalysts.

### 3.2 UV–vis Spectroscopy

UV–vis spectroscopy is widely used to quantify organonitrogen products generated in electrochemical C—N coupling reactions [69]. The diacetyl monoxime (DAMO) method enables both visual and spectroscopic verification of C—N coupled products, and the indophenol blue assay is applied to quantify ammonia, which serves as an important metric for evaluating catalytic selectivity in C—N coupling systems [70, 71].

In the DAMO method, diacetyl monoxime is converted into diacetyl under acidic, high‐temperature conditions and subsequently reacts with urea in a ferric chloride–thiosemicarbazide solution to form a characteristic reddish complex (Figure 3c). UV–vis spectra recorded at potentials from −0.3 to −0.8 V exhibited a pronounced absorption band around 525 nm (Figure 3d). This trend reflects the potential‐dependent formation of the target organonitrogen species, and the linear correlation between absorbance and concentration confirms the quantitative reliability of the DAMO‐based assay. The conventional DAMO method is commonly used to quantify urea. However, it exhibits critical limitations in electrochemical systems utilizing nitrate as the nitrogen source. The most detrimental drawback arises from its extreme susceptibility to interference by nitrite, a common reaction intermediate in nitrate reduction. Such interference makes it difficult to accurately detect urea, particularly at concentrations below 5 ppm, false‐positive signals often appear, and urea concentrations are frequently measured higher than actual levels [72]. Therefore, potential interferences should be carefully considered, and complementary verification methods should be applied whenever possible to ensure accurate and reproducible urea measurements in nitrate‐containing electrochemical systems.

Conversely, the indophenol blue method quantifies ammonia (NH_3_) *via* the formation of a blue‐colored complex under alkaline conditions [71]. Since NH_3_ often arises as a competing byproduct from the over‐reduction of nitrogen species, this colorimetric method serves as a key indicator of catalytic selectivity in C—N coupling systems [19]. In this method, ammonia reacts in the presence of sodium hypochlorite and sodium nitroprusside to generate monochloramine as a reactive intermediate. The generated chloramine subsequently couples with salicylic acid or phenol to form the indophenol blue dye, which exhibits a characteristic absorption at approximately 630–655 nm [73]. The absorbance intensity of the indophenol chromophore correlates linearly with the NH_3_ concentration, allowing for quantitative measurement through UV–visible spectrophotometry [73]. In urea synthesis, the difference in total NH_3_ concentration measured with and without urease addition can be used to estimate urea yield. Nevertheless, factors such as enzyme activity, temperature, enzyme loading, and interference from metal cations must be carefully controlled to ensure quantitative reproducibility [74].

### 3.3 HPLC

HPLC facilitates baseline separation of polar, nonvolatile C—N coupling products (e.g., urea, formamide) with quantification down to low micromolar concentrations [72]. This technique minimizes spectral overlap common in UV–vis analysis and, when coupled with UV–vis or mass spectrometric detectors, allows simultaneous identification and quantification of multiple nitrogen‐containing products such as urea, formamide, and acetamide [72].

For instance, a typical ^18^C column with acetonitrile–water mobile phase (flow rate: 0.3 mL min^−1^) and UV detection at 195 nm yields a sharp urea peak at approximately 10.8 min (Figure 3e). The peak intensity increased proportionally with urea concentration, validating both the presence and quantifiability of urea in a 0.2 M KHCO_3_ electrolyte. These results highlight HPLC as a robust tool for the reliable detection of soluble C—N coupling products. Beyazay et al. analyzed postreaction liquid products by HPLC and identified formamide and acetamide through comparison of their retention times with those of authentic standards [75]. This analysis confirmed that C—N coupled products formed in aqueous media can be reliably identified using HPLC.

### 3.4 GC–MS

Gas chromatography–mass spectrometry (GC–MS) is a highly sensitive method for identifying and quantifying volatile C—N coupling products such as amides and amines [76]. Operating in either positive chemical ionization (PCI) or electron ionization (EI) mode, GC–MS provides high‐resolution molecular and fragment ion profiles that unambiguously confirm C—N bond formation [76, 77]. Product identification is achieved through comparison with authentic standards, while quantification relies on external calibration curves [78]. Rigorous blank controls are essential to prevent cross‐contamination [38]. The GC–MS spectrum of N‐methylacetamide exhibits a prominent molecular ion peak at *m/z* = 73, with major fragment peaks at *m/z* = 29, 43, and 60 resulting from the cleavage of the molecular ion (Figure 3f).

In contrast, nonvolatile compounds such as urea and formamide are identified using liquid chromatography–mass spectrometry (LC–MS). LC–MS enables direct analysis of reaction products in solution, providing molecular ion information and structural insights without derivatization. The unique mass‐to‐charge characteristics of C—N coupling products allow efficient discrimination from undesired byproducts formed during electrochemical reactions [79, 80]. Zhou et al. applied a chromatography‐based qualitative and quantitative approach to reliably identify urea produced in the electrochemical reaction [31]. In LC–MS analysis, urea exhibited a characteristic molecular ion peak at *m/z* = 61, along with minor metallic adduct signals. Quantification was performed using calibration curves established with ^15^N‐ or ^13^C‐labeled isotopic standards, confirming that the detected urea originated from electrochemical C—N coupling rather than external contamination. This analytical protocol provides a practical and reliable framework for the accurate identification and quantification of nonvolatile C—N coupling products using LC–MS.

### 3.5 Differential Electrochemical Mass Spectrometry (DEMS)

DEMS is a powerful operando technique that enables real‐time detection of volatile reaction products during electrochemical processes [48]. By coupling an electrochemical cell with a membrane inlet connected to a mass spectrometer, DEMS allows continuous monitoring of gaseous and volatile species directly at the electrode–electrolyte interface [81]. Unlike conventional ex situ methods such as GC–MS and LC–MS, DEMS allows tracking of how products form and change in real time, enabling the correlation of product formation with applied potential or current. This feature is particularly valuable for elucidating reaction pathways and identifying transient intermediates in C—N coupling systems [82].

In DEMS analysis, product identification is based on characteristic mass‐to‐charge (*m/z*) signals. For instance, CO is typically detected at *m/z* = 28, while NO_2_ at *m/z* = 46, NO at *m/z* = 30, and NH_3_ at *m/z* = 17 (Figure 3g). The temporal evolution of these signals under controlled electrochemical conditions provides mechanistic insight into intermediate formation and consumption.

Overall, DEMS complements conventional ex situ techniques by enabling operando monitoring of volatile species and transient intermediates, thereby providing critical insight into reaction kinetics and mechanistic pathways that are not accessible through postreaction analysis alone. This makes DEMS particularly useful for resolving the dynamic behavior of key intermediates involved in C—N bond formation.

### 3.6 NMR Spectroscopy

Nuclear magnetic resonance (NMR) spectroscopy delivers definitive structural and quantitative insights into electrochemically generated C—N products. By monitoring diagnostic chemical shifts and coupling patterns, NMR complements colorimetric and chromatographic assays such as UV–vis with orthogonal validation [83, 84]. Deuterated solvents, including D_2_O or DMSO‐d_6_, are commonly utilized to minimize background proton signals through rapid hydrogen–deuterium exchange, thereby improving spectral resolution [85].

Characteristic ^1^H NMR signals unambiguously indicate the formation of target C—N coupled products. In the electrocatalytic synthesis of formamide (HCONH_2_) from CO and NO_2_^−^, the corresponding ^1^H NMR peak confirms product formation (Figure 3h). Control experiments in the sole CO reduction reaction (CORR) and nitrite reduction reaction (NO_2_RR) show no formation of HCONH_2_, demonstrating that both reactants are required for the C—N coupling reaction. Furthermore, the use of isotopically labeled precursors (e.g., ^15^N‐labeled NO_2_^−^ and ^13^C‐labeled CO) provides additional verification of the carbon and nitrogen sources. Complementary analytical techniques, such as GC–MS, can also be employed to further substantiate the product identification [60, 83].

## 4 Catalytic Design Strategies for C—N Coupled Products

The selective formation of C—N coupled products requires the simultaneous activation of both carbon and nitrogen precursors on the catalyst surface [86]. Competing pathways, such as CO_2_ reduction (CO_2_RR), nitrite/nitrate reduction (NO_*x*_RR), and hydrogen evolution, can deplete reactants and reduce the overall efficiency of C—N coupling [87]. Accordingly, rational catalyst design that tailors active sites and electronic environments is essential to promote C—N coupling while suppressing undesired side reactions. Herein, we introduce representative strategies reported in the literature, including heterostructure engineering, alloying, vacancy engineering, and single‐atom catalysts, to achieve high Faradaic efficiency and elevated product yields. Table 2 shows the summary of recently reported electrocatalysts for C—N coupled products and their performances.

**TABLE 2:** Performances of recently reported electrocatalysts for C–N coupled products.

Class of Catalyst	Catalyst	N Source	C Source	Product	**Potential,** **vs. RHE**	**FE,** **%**	Yield rate	Electrolyte	Cell type	Ref.
Heterostructure	Fe^II^‐Fe^III^OOH@BiVO_4_‐2	NO_3_^−^	CO_2_	Urea	−0.8 V	11.50	13.8 mmolh^−1^ g^−1^	CO_2_‐saturated 0.1 M KNO_3_	H‐cell	[88]
	Ru‐Cu CF	NO_3_^−^	CO_2_	Urea	−0.13 V	25.4	151.6 μg h^−1^ cm^−2^	CO_2_‐saturated 0.1 M NaNO_3_	—	[89]
	CoPc‐COF@TiO_2_ NT	NO_3_^−^	CO_2_	Urea	−0.6 V	49	1205 μg h^−1^ cm^−2^	CO_2_‐saturated KHCO_3_ + KNO_3_	Flow cell	[90]
	RP‐CuAu	NO_3_^−^	CO_2_	Urea	−0.6 V	88.5	22.9 mmol g^–1^ h^–1^	CO_2_‐saturated 0.1 M KHCO_3_ + 0.1M KNO_3_	H‐cell	[15]
	In_2_O_3_	NO_3_^−^	CO_2_	Urea	−0.35 V	10.46	357.47 μg mg ^−1^ h ^−1^	CO_2_‐saturated 0.1 M KNO_3_ + 0.1M KHCO_3_	H‐cell	[91]
	Ru1@Cu_3_N	NO_3_^−^	CO_2_	Urea	−0.7 V	52.66	39.88 mmolh^–1^ g^–1^	CO_2_‐saturated 0.1 M KHCO_3_ + 0.1 M KNO_2_	Flow cell	[16]
	FeN_4_/B_2_CuN_2_@NC	NO_3_^−^	CO_2_	Urea	−0.4 V	71.9	2072.5 µg h^−1^ mg_cat_^−1^	CO_2_‐saturated 0.1 M KHCO_3_ + 0.1 M KNO_3_	H‐cell	[31]
	Cu/Cu_2_O‐NRH	NO_3_^−^	CO_2_	Urea	−0.3 V	32.6–47.0	6.08–30.4 μmol h^−1^cm^−2^	CO_2_‐saturated 0.5M KHCO_3_ + 0.1M KNO_3_	Flow cell	[92]
	NF@CoMn_2_O_4_@ZnO‐TiO_2_	NO_2_^−^	CO_2_	Urea	−0.6 V	61.18	5.79 µg cm^−2^ h^−1^	CO_2_‐saturated 0.2 M KHCO_3_ + 0.1M KNO_2_	H‐cell	[93]
	Cu NCs/BN	NO_3_^−^	CO	Acetamide	−1.6 V	13.9	137.0 mmol h^–1^ g_cat_^–1^	CO_2_‐fed 0.02M KNO_3_ + 0.1 M KCl	Flow cell	[94]
	Co_2_Cu_1_PPC	NO_3_^−^	CO_2_	Methylamine	−0.76 V	11.30	—	CO_2_‐saturated 0.1 M KHCO_3_ + 0.8M KNO_3_	H‐cell	[53]
	CoPc‐NH_2_	NO_3_^−^	CO_2_	Methylamine	−0.79 V	12.4	0.12 μmol h^−1^ cm^−2^	—	—	[95]
	3CuO_*x*_/7BiO_*x*_	NO_2_^−^	CO_2_	Formamide	−0.3 V	—	134 ± 11 mmol h^−1^g_cat_^−1^	CO_2_‐fed 0.2M KHCO_3_ + 0.02M KNO_2_	Flow cell	[27]
Alloy	PdCu/CBC	NO_3_^−^	CO_2_	Urea	−0.5 V	69.1 ± 3.8	763.8 ± 42.8 μg h^−1^ mg^−1^	CO_2_‐saturated 0.05M KNO_3_	H‐cell	[17]
	RhCu‐uls	NO_3_^−^	CO_2_	Urea	−0.6 V	34.82 ± 2.47	26.81 ± 0.62 mmol h^−1^ g^−1^	CO_2_‐saturated 0.1M KNO_3_	H‐cell	[80]
	XC72R‐AuPd	NO_3_^−^	CO_2_	Urea	−0.5 V	15.6	204.2 μg mg^−1^ h^−1^	CO_2_‐saturated 0.075 M KHCO_3_ + 0.0.25 M KNO_3_	H‐cell	[18]
	Cu@Zn	NO_3_^−^	CO_2_	Urea	−1.02 V	9.28	7.29 μmol cm^–2^ h^–1^	CO_2_‐fed 0.2M KHCO_3_ + 0.1M KNO_3_	H‐cell	[96]
	Ru‐Cu_9_Bi/CNT	NO_3_^−^	CO_2_	Urea	−0.4 V	75.6	48.8 µmol h^–1^ mg^–1^	CO_2_‐saturated 0.1 M KHCO_3_ + 0.1M KNO_3_	H‐cell	[97]
	Pd_4_Cu_1_‐FeNi(OH)_2_	NO_3_^−^	CO_2_	Urea	−0.4 V	66.4	436.9 mmol g_cat_^−1^ h^−1^	CO_2_‐saturated 0.1 M KNO_3_	H‐cell	[98]
	CuPd_1_Rh_1_‐DAA	NO_3_^−^	CO_2_	Urea	−0.5 V	72.10	53.2 mmol h^−1^ g_cat_^−1^	CO_2_‐fed 0.1 M KHCO_3_ + 0.1M KNO_3_	H‐cell	[99]
	Cu_1_Au_8_@CeO_2_	NO_3_^−^	CO_2_	Urea	−0.74 V	45.2	813.6 μg h^−1^ mg_cat_^−1^ (@ −0.94V vs. RHE)	CO_2_‐saturated 0.1 M KNO_3_	H‐cell	[100]
Vacancy	Vo‐S‐IO‐6	NO_3_^−^	CO_2_	Urea	−0.6 V	60.6	910.4 μg h^−1^ mg^−1^	CO_2_‐saturated 0.1 M KNO_3_	H‐cell	[20]
	Vo‐InOOH	NO_3_^−^	CO_2_	Urea	−0.5 V	51	592.5 mg h^–1^ g^–1^	CO_2_‐saturated 0.1 M KNO_3_	H‐cell	[101]
	Vo‐CeO_2_‐750	NO_3_^−^	CO_2_	Urea	−1.3 V	3.84	943.6 mg h^–1^ g^–1^ (@ −1.6 V vs. RHE)	CO_2_‐saturated 0.1 M KNO_3_	H‐cell	[102]
	Vo‐In‐TiO_2_	NO_3_^−^	CO_2_	Urea	−0.65 V	9.06	759.8 μg mg^−1^ h^−1^	CO_2_‐saturated 0.1 M KHCO_3_ + 0.1 M KNO_3_	H‐cell	[103]
	Co_3_O_4_−1.0	NO_2_^−^	CO_2_	Urea	−0.7 V	26.3	3361 mg h^−1^ g_cat_^−1^	CO_2_‐saturated 0.2M KHCO_3_ + 0.02M KNO_3_	H‐cell	[104]
	ZnO‐V	NO_2_^−^	CO_2_	Urea	−0.79 V	23.26	16.56 μmol·h^−1^·cm^−2^	CO_2_‐saturated 0.2 M NaHCO_3_ + 0.1 M NaNO_3_	H‐cell	[105]
	Cu‐TiO_2_	NO_2_^−^	CO_2_	Urea	−0.4 V	43.1	20.8 μmol·h^−1^ (@ − 0.7 V vs. RHE)	CO_2_‐saturated 0.2 M KHCO_3_ + 0.02 M KNO_3_	—	[19]
Single‐atom	Cu‐GS‐800	NO_3_^−^	CO_2_	Urea	−0.9 V	28	4.3 nmol s^–1^ cm^–2^	CO_2_‐saturated 0.1 M K_2_SO_4_ + 0.1 M KNO_3_	H‐cell	[83]
	Ru_1_Cu SAA	NO_2_^−^	CO	Formamide	−0.5 V	45.65 ± 0.76	2483.77 ± 155.34 μg h^−1^ mg_cat_^−1^	CO_2_‐saturated 1 M KOH + 1 M KNO_2_	H‐cell	[45]
	FeNC‐Fe_1_N_4_	NO_3_^−^	CO_2_	Urea	−0.6 V	66.50	38.2 mmol g_cat_^−1^ h^−1^ (@ – 0.9 V vs. RHE)	CO_2_‐saturated 0.1 M KHCO_3_ + 0.1 M KNO_3_	H‐cell	[106]
	Co_1_‐TiO_2_	NO_3_^−^	CO_2_	Urea	−0.8 V	36.20	—	CO_2_‐saturated 0.1 M K^+^	H‐cell	[107]
	L‐Cu_1_‐CeO_2_	NO_3_^−^	CO_2_	Urea	−1.6 V	—	52.84 mmol h^−1^ g_cat_^−1^	CO_2_‐saturated 0.1 M KHCO_3_ + 0.05 M KNO_3_	H‐cell	[21]
	CoPC‐NH_2_/CNT	NO_3_^−^	CO_2_	Methylamine	−0.92 V	13	—	CO_2_‐saturated 0.1 M KHCO_3_ + 0.5M KNO_3_	H‐cell	[51]

### 4.1 Heterostructure Engineering

Heterostructure engineering is widely used in electrochemical C—N coupling by constructing intimate interfaces between dissimilar materials, thereby inducing synergistic effects, charge redistribution, and selective intermediate adsorption [108]. The intimate contact between heterogeneous components facilitates interfacial charge transfer, modulating the local electronic structure and strengthening adsorption of key intermediates such as ^*^CO and ^*^NH_2_ [38, 68]. Moreover, engineered interfaces can improve electron and mass transport, suppress side reactions, and enhance catalytic stability [109]. Precise control over composition, interface density, and morphology is thus critical for high‐efficiency synthesis of C—N bond–containing species. Nevertheless, the complexity of synthesis protocols and potential interfacial instability remain challenges for reproducibility and long‐term durability [110, 111].

**FIGURE 4:**
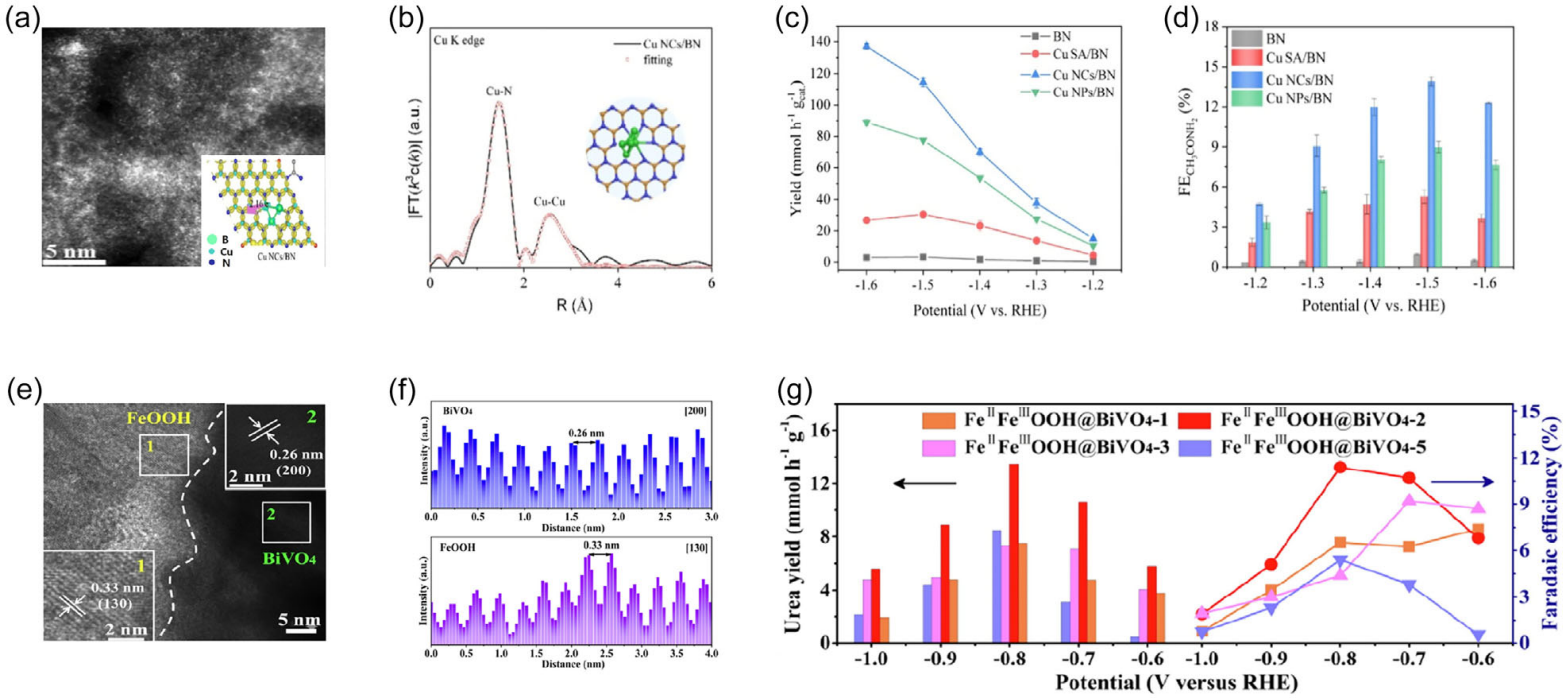
(a) High‐resolution transmission electron microscopy (HR‐TEM) image of Cu NCs/BN (inset: corresponding atomic structure model). (b) Cu K‐edge EXAFS spectra of Cu NCs/BN. (c) Potential‐dependent product yields. (d) FE toward acetamide formation over BN, Cu SA/BN, Cu NCs/BN, and Cu NPs/BN catalysts. Adapted with permission from Wang et al. [94]. Copyright 2025, ACS. (e) HR‐TEM image illustrating the heterointerface between FeOOH and BiVO_4_. (f) Lattice spacing analysis derived from the intensity line profiles of image (e). (g) Potential‐dependent yield and FE for urea synthesis over various FeOOH@BiVO4 heterostructure catalysts. Adapted with permission from Yin et al. [88]. Copyright 2025, Elsevier.

The effectiveness of this approach is illustrated by several tailored heterostructure designs. For instance, the integration of Cu nanoclusters with boron nitride nanosheets creates an atomic‐scale catalytic interface. Wang et al. demonstrated that Cu nanoclusters supported on boron nitride nanosheets (Cu NCs/BN) serve as highly efficient atomic‐scale catalysts for acetamide production [94]. High‐angle annular dark‐field scanning transmission electron microscopy (HAADF–STEM) images clearly show that the Cu nanoclusters are homogeneously dispersed on the BN support with near‐atomic resolution (Figure 4a). The fitted Cu K‐edge extended X‐ray absorption fine structure (EXAFS) spectra further reveal a distinct coordination environment compared to bulk Cu foil and Cu oxides, suggesting strong electronic interactions between the Cu clusters and the BN substrate (Figure 4b). This interfacial electronic modulation effectively enhances the activation and adsorption of key intermediates (e.g., ^*^CCO and ^*^NH_2_), thus facilitating C—N bond formation. Consequently, the Cu NCs/BN catalyst delivers a remarkable acetamide yield rate of 137.0 mmol h^−1^ g_cat_^−1^ at −1.6 V (vs. RHE), along with a high Faradaic efficiency even under elevated current densities (Figure 4c). In addition, potential‐dependent Faradaic efficiency (FE) measurements confirm that the Cu NCs/BN heterostructure exhibits enhanced stability and selectivity for C—N coupling compared to its single‐atom (CuSA/BN) and nanoparticle (CuNPs/BN) counterparts (Figure 4d).

Constructing heterojunctions with interfacial synergistic effects using metal oxides/hydroxides can also promote complex coreduction reactions. For instance, Zhang et al. demonstrated that the Fe^2+^–Fe^3+^OOH nanoparticles anchored on BiVO_4_ (FeOOH@BiVO_4_) synergistically promote urea synthesis from CO_2_/NO_3_^–^ [88]. HR‐TEM images reveal that Fe^2+^–Fe^3+^OOH nanoparticles are uniformly anchored on the BiVO_4_ surface, with distinct lattice spacings of 0.33 nm (FeOOH) and 0.26 nm (BiVO_4_) (Figure 4e). Line‐scanning intensity profiles further confirm the intimate interfacial contact between Fe^2+^–Fe^3+^OOH and BiVO_4_ domains, verifying the successful construction of the heterostructured interface (Figure 4f). Benefiting from this well‐defined heterostructure, Fe^2+^–Fe^3+^OOH @BiVO_4_−2 exhibits a high urea yield rate of 13.8 mmol h^−1^ g^−1^ and a Faradaic efficiency of 11.5% at −0.8 V (vs. RHE) (Figure 4g). Such enhancement is attributed to the synergistic coupling between Fe redox pairs and the BiVO_4_ semiconductor, which facilitates charge transfer and stabilizes the C—N coupling intermediates at the interface.

Furthermore, metal oxides are widely applied to construct heterostructures. A hollow cubic heterostructure composed of CuO and In2O3, reported by Shen's group, exemplifies the design of dual‐site catalytic systems. Shen et al. designed a CuO/In_2_O_3_ hollow cube heterostructured catalyst to efficiently promote the electrochemical synthesis of formamide through interfacial electron interactions [112]. HR‐TEM revealed that CuO and In_2_O_3_ are assembled into a well‐defined hollow cubic framework, forming an intimate interface between the two phases. X‐ray photoelectron spectroscopy (XPS) analysis and electron density difference mapping further confirmed charge redistribution across the CuO–In_2_O_3_ junction, indicating strong interfacial electronic coupling. Such interfacial interaction enables the CuO domain to preferentially activate carbon‐containing intermediates, while the In_2_O_3_ domain facilitates the activation of nitrogen‐containing intermediates, thereby promoting C—N bond formation through a dual‐site synergistic mechanism. DFT calculations demonstrated that this dual‐site pathway effectively lowers the reaction energy barrier and accelerates the CO–NH_2_ coupling step compared to single‐phase catalysts. As a result, the CuO/In_2_O_3_ heterostructure achieved an outstanding formamide yield rate of 8.43 ± 1.54 g h^−1^ mg_cat_^−1^ and a maximum FE of approximately 49%, significantly outperforming the individual In_2_O_3_ nanorods (27.85%) and CuO cubes (14.58%). Overall, this work underscores that heterostructure engineering, by modulating interfacial charge transfer and creating dual active sites, provides an effective design strategy for enhancing electrochemical C—N coupling toward organic nitrogen compounds.

In summary, heterostructure engineering enhances electrochemical catalytic C—N bond formation by facilitating interfacial charge transfer and establishing dual active sites. Through the synergistic interaction between distinct components, these heterostructures promote simultaneous activation of intermediates, aiding C—N bond formation. However, the complex synthesis process and potential interfacial instability remain key challenges, highlighting the need for more controllable and stable heterostructure designs in future catalyst development.

### 4.2 Alloy Engineering

In recent studies on electrochemical C—N coupling between CO_2_ and NO_*x*_^–^, alloying has attracted considerable attention as an effective catalyst design strategy [113]. By adjusting the electronic structure and geometric arrangement of active sites, alloy catalysts can improve both the activity and selectivity toward urea and other organonitrogen products [43, 64]. In addition, tuning the alloy composition helps to optimize the adsorption energies of key intermediates and to suppress competing reactions such as the hydrogen evolution reaction (HER) [98]. Therefore, precise control over metal combinations, alloy composition, and structural integration is crucial for developing efficient and durable catalysts for high‐value organonitrogen synthesis.

**FIGURE 5:**
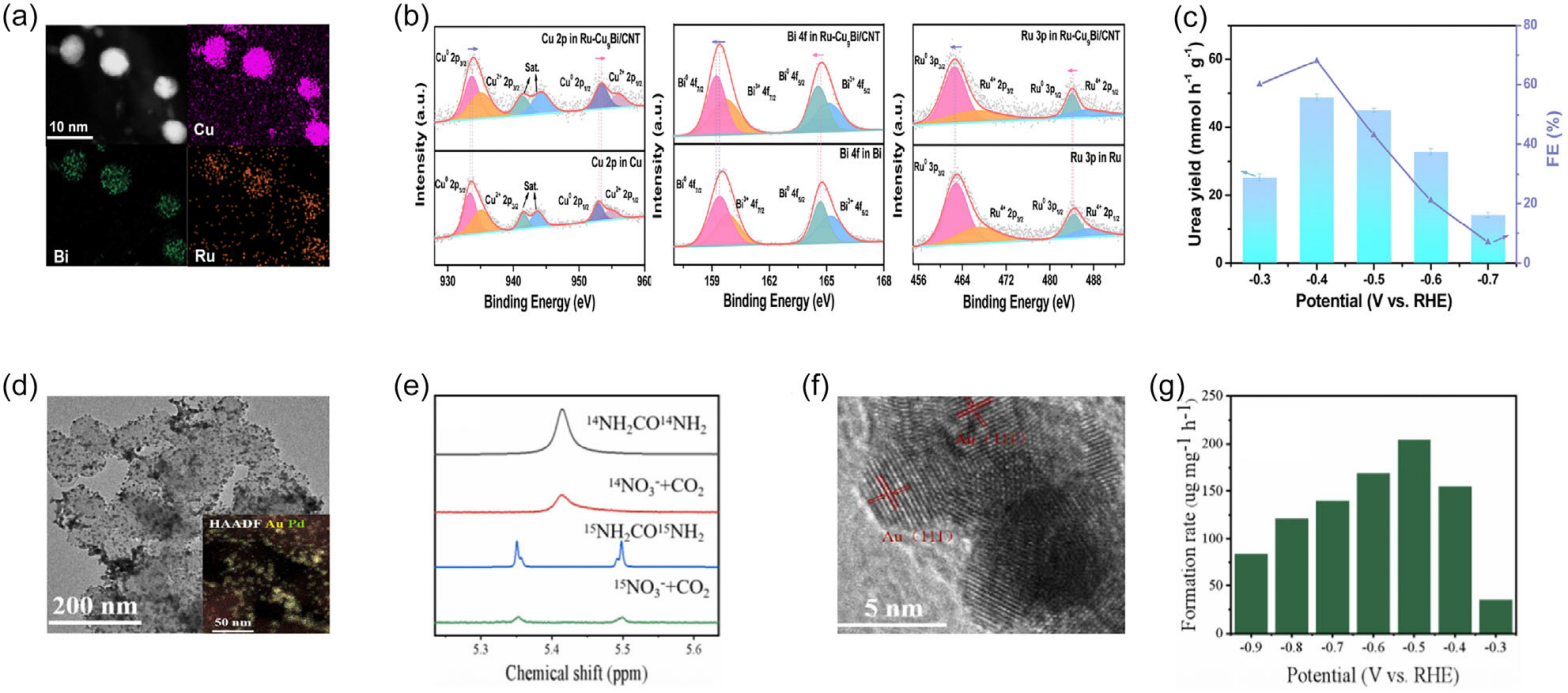
(a) HAADF–STEM image of Ru‐Cu/CNT and the EDS images of Cu, Bi, and Ru. (b) Cu 2p, Bi 4f, and Ru 3p XPS spectra between Ru‐Cu‐Bi/CNT and single‐metal reference samples. (c) Potential‐dependent urea yield and FE. Adapted with permission from Yu et al. [97]. Copyright 2025, RSC. (d) TEM image of Ag‐Pd nanoparticles (inset” corresponding HAADF–STEM image). (e) 1H NMR spectra confirming urea formation *via* isotope‐labeling experiments. (f) HR‐TEM image of Au/Pd nanoparticles. (g) Potential‐dependent formation rate of urea. Adapted with permission from Wang et al. [18]. Copyright 2025, Elsevier.

The effectiveness of alloying engineering has been demonstrated in various multimetallic catalyst systems. By introducing additional metal components into Cu‐based catalysts, atomic‐scale alloy interfaces can be formed, allowing fine‐tuning of electronic interactions and surface reactivity. Yu et al. developed a Ru‐Cu_9_Bi/CNT catalyst by partially alloying Ru into Cu‐Bi, aiming to fine‐tune the electronic interactions among multiple metals and thereby promote the electrochemical C—N coupling between CO_2_ and NO_3_^–^ [97]. Elemental mapping images clearly show that Cu, Bi, and Ru are homogeneously distributed on the carbon nanotube (CNT) support, suggesting that the three metals are uniformly alloyed at the atomic level to create multiple active sites (Figure 5a). The Cu 2p XPS spectra further reveal a noticeable shift in binding energy compared to pure Cu, implying that the electronic density of Cu was effectively tuned through the electronic interplay with Ru and Bi (Figure 5b). This alloying‐induced modification in the electronic structure optimizes the adsorption and activation of intermediates during both CO_2_ reduction and NO_3_^−^ reduction, thus favoring the C—N bond formation. As a result, the Ru–Cu_9_Bi/CNT catalyst delivers a remarkable urea yield rate of 40.0 mmol h^−1^ g^−1^ and a Faradaic efficiency of 75.6% at –0.4 V (vs. RHE), significantly outperforming the monometallic counterparts (Figure 5c).

Alloying Au with Pd has been shown to optimize the electronic configuration relative to the corresponding monometallic catalysts, thereby enhancing catalytic performance in complex coreduction reactions. Wang et al. synthesized Au–Pd alloy nanoparticles supported on Vulcan XC72R carbon to achieve the simultaneous electroreduction of CO_2_ and NO_3_^−^ [18]. TEM images confirmed that the nanoparticles were uniformly dispersed on the carbon support, exhibiting a well‐defined hierarchical nanostructure (Figure 5d). The ^1^H NMR spectra of standard urea samples (^14^NH_2_CO^14^NH_2_ and ^15^NH_2_CO^15^NH_2_) were compared with those of the electrolytes obtained after the coreduction of CO_2_ and NO_3_^−^. When ^15^NO_3_^−^ was used as the nitrogen source, distinct ^15^N‐labeled signals were observed, strongly evidencing that the produced urea originated from both CO_2_ and NO_3_^−^ (Figure 5e). High‐resolution TEM images further revealed that Au and Pd atoms were homogeneously mixed at the atomic level in the XC72R–AuPd–10% catalyst, confirming the successful formation of an alloyed structure that allows effective electronic and structural tuning between the two metals (Figure 5f). The catalytic performance, evaluated across a range of applied potentials, showed that the Au–Pd alloy achieved the highest urea yield and Faradaic efficiency around –0.5 V (vs. RHE) (Figure 5g). These findings indicate that alloying between Au and Pd optimizes the electronic configuration compared to their monometallic counterparts, facilitating the formation of key reaction intermediates and generating multiple active sites that favor C–N bond coupling.

Moreover, alloying engineering can effectively reduce the energy barrier for C—N coupling compared to conventional monometallic systems. Lan et al. designed atomically dispersed Ru—Cu nanocluster alloys (Ru_1_Cu single‐atom alloys) to promote C—N bond formation through the simultaneous electroreduction of CO and NO_2_^−^, enabling the selective synthesis of formamide [45]. In this alloyed structure, isolated Ru atoms were incorporated into or substituted within the Cu nanocluster lattice, forming adjacent multiactive sites with Cu that effectively modulated the local electronic structure and adsorption environment compared to monometallic counterparts. Structural characterizations, including HAADF–STEM and elemental mapping, confirmed that Ru atoms were homogeneously dispersed throughout the Cu matrix, while XPS and X‐ray absorption spectroscopy (XAS) analyses revealed distinct charge transfer and coordination changes between Ru and Cu atoms. This alloyed Ru–Cu configuration facilitated the concurrent generation and spatial proximity of ^*^CO and ^*^NH_2_ intermediates, favoring their coupling toward C—N bond formation. Electrochemical measurements showed that the catalyst achieved a high Faradaic efficiency of 45.65% and a formamide yield rate of 2483.8 μg h^−1^ mg_cat_^−1^ at –0.5 V vs. RHE. Moreover, in situ spectroscopic studies and DFT calculations demonstrated that ^*^CO and ^*^NH intermediates readily coupled under a low activation barrier to form the final HCONH_2_ product. This pathway effectively lowered the C—N coupling energy barrier compared to conventional monometallic systems, highlighting alloy engineering as a powerful strategy to enhance catalytic activity and selectivity.

In summary, the alloying strategy serves as an effective approach to precisely design the electronic structure and create multiple active sites by integrating two or more metals at the atomic level. Such modulation of electronic states optimizes the adsorption of key intermediates involved in C—N bond formation, thereby enhancing the catalytic performance toward C—N coupled products. However, alloy catalysts are prone to phase segregation and surface reconstruction under reaction conditions, potentially leading to instability of active sites and decreased catalytic performance [98]. Furthermore, precise control over alloy composition and atomic distribution remains challenging, highlighting the need for effective stabilization strategies during synthesis [114, 115].

### 4.3 Vacancy Engineering

Vacancies in electrocatalysts act as active sites, promoting the coadsorption and activation of reactant precursors [20]. These adsorbed species then undergo PCET processes. During this stepwise reaction, the adsorbed species are converted into key intermediates such as ^*^CO, ^*^NH_2_, and ^*^CONH, which subsequently couple to form various C—N‐bonded products [116]. In addition, the introduction of vacancies is recognized as an effective strategy to modulate the electronic structure, optimize reaction kinetics, and enhance electrical conductivity [117]. Such electronic reconstruction tunes the adsorption energy of reaction intermediates, preventing excessive binding or insufficient interaction, and thereby promotes selective C—N coupling [117]. Consequently, electrocatalysts with well‐engineered vacancy structures can achieve a delicate balance among reactant adsorption, activation, charge transfer, and intermediate coupling, ultimately enabling the efficient synthesis of diverse C—N coupled products [20, 105].

**FIGURE 6:**
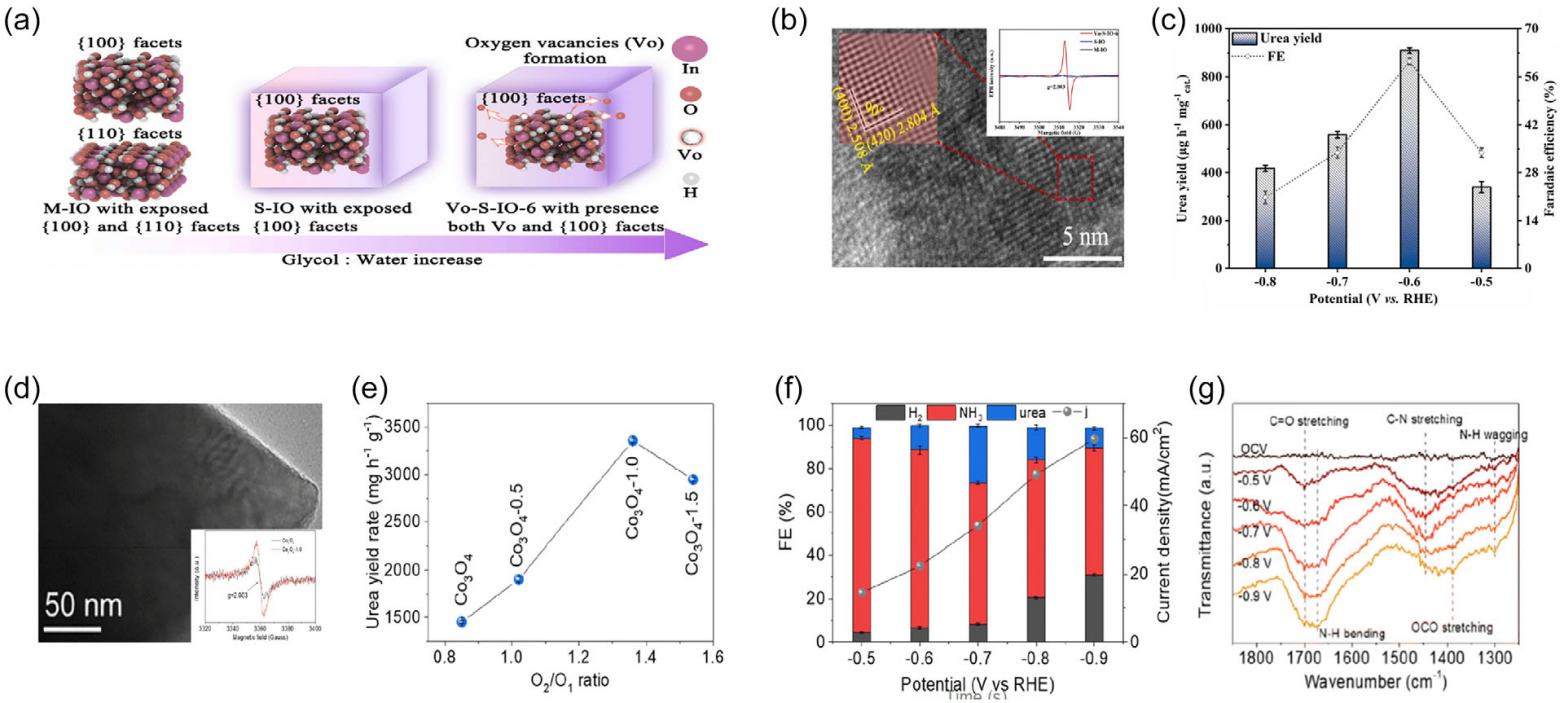
(a) Schematic illustration of the synthesis of In_2_O_3_. (b) HR‐TEM image of Vo‐S‐IO‐6 (inset: EPR spectrum confirming the presence of oxygen vacancies). (c) Potential‐dependent urea yield and FE. Adapted with permission from Li et al. [20]. Copyright 2025, Elsevier. (d) TEM image of the Co_3_O_4_ catalyst (inset: corresponding EPR spectrum). (e) Correlation between the oxygen vacancy ratio (O_V_/O_I_) and the urea yield rate. (f) Potential‐dependent FE. (g) In situ IR spectra collected at various applied potentials, showing the C—N coupling intermediates during the reaction. Adapted with permission from Li et al. [104]. Copyright 2025, RSC.

Coupling vacancy engineering with surface facet regulation provides an effective means to stabilize key intermediates and suppress competing reaction pathways. Li et al. synthesized indium hydroxide (In(OH)_3_) nanocube catalysts (Vo‐S‐IO‐6) by introducing oxygen vacancy (V_o_) to enhance the efficiency of electrochemical urea synthesis from CO_2_ and NO_3_^−^ [20]. During solvothermal synthesis, they tuned the glycol content to control the exposed crystal facets, obtaining well‐defined nanocubes predominantly enclosed by the {100} plane (Figure 6a). By further adjusting the glycol/water ratio, a series of samples with different oxygen‐vacancy concentrations (Vo‐S‐IO‐n) was obtained. Morphological and structural characterizations revealed that Vo‐S‐IO‐6 maintained a uniform nanocubic morphology with dominant {100} facet exposure, as confirmed by HR‐TEM (Figure 6b). Electron paramagnetic resonance (EPR) spectroscopy exhibited a characteristic *g* = 2.003 signal, confirming the presence of oxygen vacancies (Figure 6b). Electrochemical tests were carried out in a CO_2_‐saturated 0.1 M KNO_3_ electrolyte over a potential range from −0.5 to −0.8 V (vs. RHE) to evaluate the catalytic performance for urea synthesis. Both the urea yield rate and FE exhibited a volcano‐type dependence on the applied potential, reaching their maxima at −0.6 V (vs. RHE) with values of 910.4 μg h^−1^ mg_cat_^−1^ and 60.6%, respectively (Figure 6c). These superior performances were attributed to the synergistic effect of facet control and oxygen‐vacancy introduction, which induced electronic reconstruction, thereby stabilizing ^*^NO_2_ and ^*^CO_2_ intermediates and promoting C–N coupling. In conclusion, the coexistence of the {100} facet and oxygen vacancies effectively directed the selective coupling between ^*^CO_2_ and ^*^NO_2_ intermediates, suppressing competing protonation pathways and achieving both high urea yield and Faradaic efficiency.

Similarly, Li et al. applied defective Co_3_O_4_ catalysts for electrochemical urea synthesis from CO_2_ and NO_2_^−^ [118]. They varied the density of oxygen vacancies, and EPR spectra revealed an enhanced signal intensity with increasing defect concentration, confirming the successful introduction of vacancies (Figure 6d). XPS O 1s analysis allowed quantitative comparison of the defect levels through the O_2_/O_1_ ratio, where a higher ratio corresponded to a greater vacancy density (Figure 6e). Among the various Co_3_O_4_ samples, Co_3_O_4_−1.0, possessing the most favorable O_2_/O_1_ ratio, exhibited the highest C—N coupling activity and was therefore selected as the optimal catalyst. Electrochemical measurements demonstrated that at −0.7 V (vs. RHE), both the urea yield rate and FE reached their maximum values, indicating that this potential was optimal for urea generation (Figure 6f). Furthermore, in situ ATR‐FTIR spectroscopy was conducted to verify the actual formation of C—N bonds during the reaction (Figure 6g). The spectra revealed distinct absorption bands corresponding to C—N stretching vibrations in intermediates derived from CO_2_ and NO_2_^−^, whose intensity and position varied with applied potential, providing direct spectroscopic evidence for the formation of urea. Collectively, these results suggest that oxygen vacancies function as active electronic modulation sites that stabilize CO_2_^–^ and NO_2_^–^‐derived intermediates and facilitate C—N coupling.

Wei et al. explored the use of cerium oxide nanorods rich in oxygen vacancies for electrochemical C–N coupling toward urea synthesis, carefully examining how these vacancies influence C—N coupling activity [102]. They synthesized CeO_2_ nanorods with tunable oxygen vacancy densities through controlled thermal treatments and employed EPR, XPS, and *in situ* sum‐frequency generation (SFG) spectroscopy to investigate how increasing vacancy concentration affects the adsorption and stabilization of intermediates. Among the samples, Vo‐CeO_2_−750, which featured the highest vacancy density, emerged as the optimal catalyst, reaching a maximum urea production rate of 943.6 mg h^−1^ g^−1^. Moreover, in situ SFG spectroscopy reveals that increasing the oxygen vacancy concentration leads to a monotonic increase in the intensity of the OCNO vibrational signal, which directly correlates with higher C—N coupling current density and product selectivity. This clear quantitative correlation demonstrates a structure–activity relationship, proving that oxygen vacancies promote C—N bond formation by stabilizing the OCNO intermediate. Overall, these results highlight the promise of vacancy engineering as a design strategy that can simultaneously enhance intermediate stabilization and bond activation in electrochemical C—N coupling, offering guidance for the development of next‐generation catalysts.

In summary, defect engineering serves as a powerful approach that simultaneously promotes the adsorption and activation of carbon and nitrogen feedstocks while stabilizing key C—N coupling intermediates to enhance product formation. Nevertheless, precise control over defect concentration and distribution remains a challenging task [119]. Furthermore, defect introduction can compromise long‐term catalyst stability, as highly defective active sites may undergo structural reconstruction under reaction conditions [120]. Therefore, developing strategies to control defect formation while maintaining catalyst stability is essential for advancing defect‐based electrocatalysts for C—N coupled product synthesis.

### 4.4 Single‐Atom Catalysts

Single‐atom catalysts (SACs), in which isolated metal atoms are stabilized on suitable supports, represent a promising strategy for maximizing atomic utilization and tuning catalytic properties at the atomic scale [121]. Because of their well‐defined coordination environments, individual metal atoms can precisely modulate the local electronic structure, thereby enabling fine control over the adsorption and activation of reactant molecules [122]. However, under reductive electrochemical conditions, SACs often suffer from metal atom migration and aggregation, as well as intrinsically low active‐site density, which limit their practical applicability [118, 123]. Hence, rational design of advanced support structures or anchoring strategies is essential to simultaneously enhance the stability of atomically dispersed sites and increase the overall density of active centers.

**FIGURE 7:**
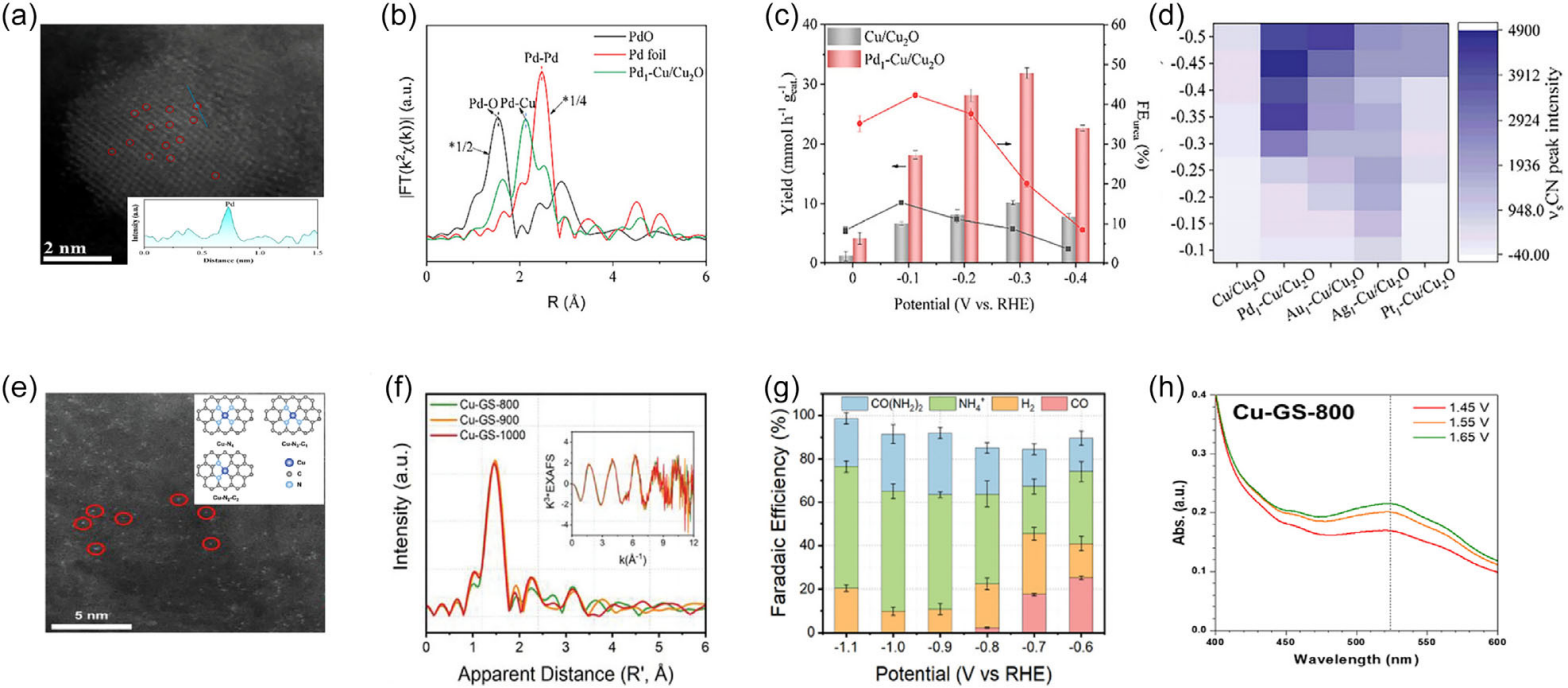
(a) HAADF–STEM image of PD_1_–Cu/Cu_2_O, where isolated Pd single atoms are highlighted with red dashed circles (inset: corresponding intensity profile). (b) Comparison of Pd K‐edge EXAFS spectra for PdO, Pd foil, and Pd_1_–Cu/Cu_2_O. (c) Potential‐dependent product yield and FE over Cu/Cu_2_O and Pd_1_–Cu/Cu_2_O catalysts. (d) Potential‐dependent ν(CN) vibrational intensity peak. Adapted with permission from Wang et al. [124]. Copyright 2025, Wiley‐VCH GmbH. (e) HAADF–STEM image of the Cu‐GS‐800 (inset: structural models of Cu–N_4_ and Cu–N_3_ coordination environments). (f) Cu K‐edge EXAFS spectra of Cu‐GS samples. (g) Potential‐dependent FE distributions for CO(NH_2_)_2_, NH_3_, H_2_, and CO. (h) In situ UV–vis absorption spectra of Cu‐GS‐800. Adapted with permission from Leverett et al. [83]. Copyright 2025, Wiley‐VCH GmbH.

In particular, introducing isolated Pd atoms in coordination with Cu sites has been shown to significantly lower the energy barrier for ^*^CO–^*^NH_2_ coupling, highlighting the potential of interface‐anchored single‐atom configurations. Wang et al. developed a Pd_1_‐Cu/Cu_2_O catalyst through the in situ electrochemical reconstruction of Pd_1_‐Cu_2_Te nanosheets, leading to single Pd atoms bridged at the Cu/Cu_2_O interface [124]. In the AC‐HAADF–STEM images, isolated bright spots corresponding to individual Pd atoms were clearly observed on the Cu/Cu_2_O support, confirming the successful formation of atomically dispersed Pd sites (Figure 7a). The Pd K‐edge Fourier transform extended X‐ray absorption fine structure (FT‐EXAFS) spectrum further revealed distinct Pd–O and Pd–Cu coordination peaks, while no Pd–Pd metallic bond was detected (Figure 7b). These results indicate that single Pd atoms are anchored at the Cu/Cu_2_O interface, forming a unique Pd–Cu dual‐site structure. To understand how this atomic configuration affects C—N coupling, the urea synthesis performance was evaluated. Compared with the Cu/Cu_2_O support, Pd_1_–Cu/Cu_2_O delivered a much higher activity, achieving a maximum FE of 42.2% at −0.1 V (vs. RHE) and a urea yield of 31.8 mmol h^−1^ g_cat_^−1^ at −0.3 V (vs. RHE) (Figure 7c). In situ Raman spectra provided further insight into the reaction pathway, revealing that Pd1‐Cu/Cu_2_O exhibited the strongest C—N bond vibration intensity among various single‐atom catalysts, including Au, Ag, and Pt analogues (Figure 7d). This suggests that the Pd–Cu dual sites promote a better balance between ^*^CO and ^*^NH_2_ intermediates by tuning the reduction kinetics of CO_2_ andNO_3_^–^. DFT calculations support this conclusion, showing that the Pd–Cu coordination lowers the coupling energy barrier of ^*^CO and ^*^NH_2_ from 1.13 eV on Cu/Cu_2_O to 0.67 eV, thereby facilitating the C—N bond formation process. Furthermore, the in situ UV–vis spectra clearly reveal that the active sites of the CU‐GS‐800 single‐atom catalyst maintain a Cu^2+^ oxidation state under C—N coupling reaction conditions.

Metals with electron‐deficient characteristics can independently act as effective active sites for C—N bond formation reactions. Leverett et al. investigated the role of single‐atom copper catalysts in the simultaneous electrochemical reduction of CO_2_ and NO_3_^–^ toward urea synthesis [83]. The authors synthesized Cu—N—C catalysts by anchoring atomically dispersed Cu atoms onto N‐doped graphene supports, followed by pyrolysis at different temperatures (800°C, 900°C, 1000°C) to precisely tune the local coordination environment of Cu sites. This temperature‐controlled synthesis strategy enabled the gradual transformation of the active site structure as the pyrolysis temperature increased. Among the prepared catalysts, the Cu–GS–800 sample, dominated by the Cu–N_4_ coordination configuration, exhibited the highest catalytic activity for urea production. HAADF–STEM images revealed that the Cu atoms were atomically dispersed as isolated bright spots without the formation of metallic nanoparticles (Figure 7e). Furthermore, Cu K‐edge FT‐EXAFS spectra of all samples showed a dominant Cu—N peak at approximately 1.5 Å and no Cu—Cu metallic coordination, confirming the successful formation of atomically dispersed Cu sites (Figure 7f). During electrochemical tests, the Cu–GS–800 catalyst achieved a remarkable FE of 28% for urea synthesis at −0.9 V (vs. RHE) (Figure 7g). This superior performance was attributed to the well‐defined Cu–N_4_ coordination, which effectively suppressed competing side reactions such as NH_4_^+^ and H_2_ formation while selectively promoting C—N bond coupling (Figure 7h). This finding indicates that the electron‐deficient Cu^2+^ centers play a crucial role in facilitating the adsorption of CO_2_‐ and NO_3_^–^‐derived intermediates and mediating the subsequent C—N bond formation.

Furthermore, the single atom‐based cooperative catalytic structure integrating the SAC and auxiliary active sites can induce the C—N bond formation pathway by maintaining a continuous supply of the key intermediate. Li et al. designed a dual‐site catalyst (FeNC‐Fe_1_N_4_) consisting of atomically dispersed Fe single atoms and metallic Fe clusters immobilized on a nitrogen‐doped carbon (NC) support [106]. In this system, the Fe_1_N_4_ single‐atom sites serve as the primary catalytic centers, exhibiting exceptional selectivity and activity for the two‐electron reduction of CO_2_ to ^*^CO. Meanwhile, the adjacent Fe clusters complement these single‐atom sites by facilitating the multielectron reduction of NO_3_^–^ to ^*^NH_2_, thereby supplying nitrogen‐based intermediates. This cooperative interplay highlights the dominant role of SACs in steering the overall C–N coupling pathway, with the clusters serving as auxiliary sites that maintain intermediate availability. HAADF–STEM images reveal the coexistence of atomically dispersed Fe atoms and larger Fe clusters on the NC support, and XAS/EXAFS analyses confirm that the Fe single atoms adopt a Fe–N_4_ coordination structure. These well‐defined Fe_1_N_4_ sites stabilize reaction intermediates within their local environment, promoting efficient C—N bond formation. As a result, the FeNC–Fe_1_N_4_ catalyst achieves an outstanding FE of 66.5% at a low potential of –0.6 V (vs. RHE) and a high urea yield rate of 38.2 mmol g_cat_^−1^ h^−1^ at –0.9 V (vs. RHE), demonstrating the remarkable synergistic effect between single‐atom and cluster sites.

In summary, SACs immobilize metal active sites on supports at the atomic scale, providing highly accessible catalytic centers for C–N coupling reactions. By precisely tuning the local coordination environment, the adsorption and activation of carbon and nitrogen feedstocks can be optimized, thereby enhancing the formation of C—N coupled products. The cooperative interactions between isolated metal atoms and the support further modulate the electronic structure, guiding reaction pathways toward more efficient C—N bond formation. Collectively, these SACs demonstrate exceptional activity and selectivity, highlighting their potential as advanced electrocatalysts for electrochemical C—N bond synthesis [125].

## 5 Other reaction engineering strategies for C—N coupling

Recent advances in electrochemical C—N coupling increasingly highlight the critical role of reaction engineering strategies in governing product selectivity and reaction efficiency. While rational catalyst design determines the intrinsic activity and adsorption properties of active sites, the overall C—N coupling performance is also strongly influenced by the dynamic reaction environment, including potential modulation, reactant supply, and interfacial charge distribution. In particular, competing pathways such as CO_2_ reduction, NO_*x*_^–^ reduction, and hydrogen evolution can be effectively regulated by controlling reaction conditions, thereby improving the availability and coupling probability of key intermediates. Accordingly, advanced reaction engineering approaches, such as oxidation‐driven pathways and pulsed electrolysis, have emerged as powerful strategies to modulate reaction kinetics and steer C—N bond formation. In this section, we summarize recent progress in these approaches and discuss their roles in enhancing the efficiency and selectivity of C—N coupled product synthesis.

### 5.1 Oxidation‐Driven C—N Coupling Pathways

While conventional C—N coupling typically proceeds via the coreduction of CO_2_ and NO_*x*_^–^ species, oxidation‐driven pathways offer an alternative route in which carbon‐containing molecules such as methanol, formic acid, and formaldehyde are partially oxidized to generate reactive intermediates [126, 127]. These intermediates can subsequently couple with nitrogen species formed under anodic potentials to yield nitrogen‐containing organic compounds [112]. Recently, electro‐oxidative C—N coupling has emerged as a promising strategy, as oxidation reactions provide finer control over reaction kinetics and product selectivity compared with reductive systems. Moreover, precise tuning of the interfacial electronic structure of catalysts enables the modulation of charge redistribution and intermediate adsorption behavior, thereby steering the C—N coupling pathway with high efficiency [112].

**FIGURE 8:**
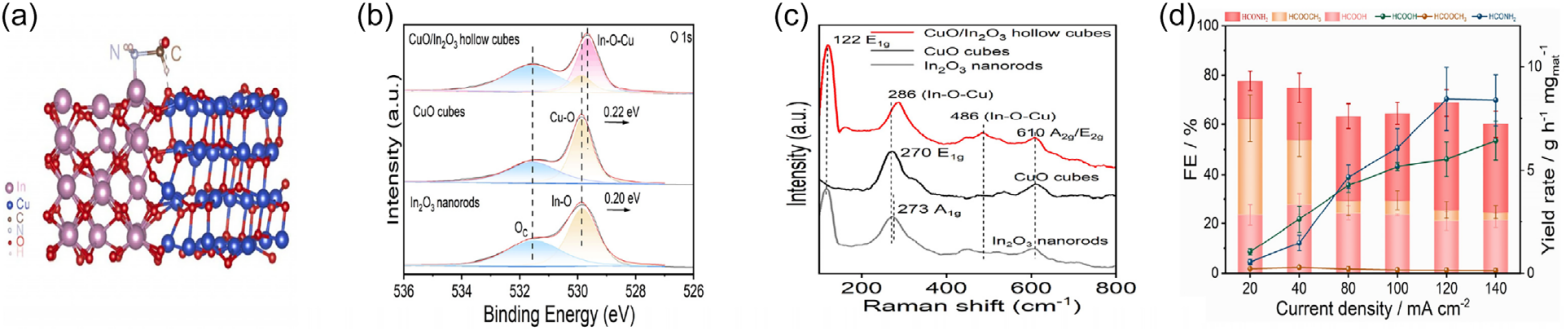
(a) Schematic illustration of the C—N coupling reaction occurring at the Cu/In–O–Cu interface. (b) O 1s XPS spectra of Cu/In–O–Cu hollow cubes, Cu_2_O cubes, and In_2_O_3_ nanorods. (c) Raman spectra of the corresponding catalysts. (d) FE and yield rate of CH_3_CONH_2_ and related products over the Cu/In–O–Cu hollow cube catalyst. Adapted with permission from Shen et al. [112]. Copyright 2025, Elsevier.

Shen et al. employed Cu/In_2_O_3_ hollow cube catalysts to synthesize formamide from methanol and aqueous ammonia [112]. The reaction mechanism was first elucidated through DFT calculations, which revealed that electrons are transferred from In sites to Cu sites via the formation of In–O–Cu interfacial bridges (Figure 8a). As a result, the ^*^CHO intermediate, generated from methanol oxidation, preferentially adsorbs on the electron‐rich Cu sites, while the NH_2_ intermediate is stabilized on the In sites. This site‐specific adsorption behavior arising from interfacial charge redistribution was further supported by O 1s XPS analysis (Figure 8b). The lattice oxygen peak of CuO/In_2_O_3_ exhibited a noticeable shift toward lower binding energy compared to the pure components, suggesting enhanced electron transfer through the In–O–Cu interface. Moreover, a distinct vibrational peak at 486 cm^−1^, observed exclusively in the CuO/In_2_O_3_ sample, serves as compelling evidence for the formation of the In–O–Cu bonding configuration (Figure 8c). These interfacial electronic interactions translated into superior electrocatalytic oxidation performance. Owing to the efficient interfacial engineering strategy, the CuO/In_2_O_3_ catalyst achieved a high FE of 49.05% for formamide production at a current density of 120 mA cm^–2^ (Figure 8d). This work demonstrates that tailoring interfacial electron transfer effectively governs the dual oxidation and subsequent C–N coupling processes, offering a powerful approach to optimize multicomponent electrocatalytic systems.

In summary, oxidation‐driven C—N coupling pathways provide an alternative reaction route that differs fundamentally from conventional coreduction systems by enabling the generation of reactive carbon intermediates via anodic processes. This approach allows more flexible control over intermediate formation, adsorption behavior, and reaction kinetics through modulation of the interfacial electronic structure. By decoupling carbon activation from purely reductive pathways, oxidation‐driven strategies can enhance the coupling probability of key intermediates and improve product selectivity. These findings highlight the potential of oxidation‐assisted pathways as an effective reaction engineering strategy for advancing the efficiency, selectivity, and controllability of electrochemical C—N bond formation.

### 5.2 Pulsed Electrolysis for C—N Coupling

Recently, pulsed electrocatalysis has emerged as a promising strategy to enhance both the reaction rate and selectivity in electrochemical processes. Unlike conventional constant‐potential electrolysis, this method periodically modulates the applied potential, thereby enabling dynamic control over the interfacial reaction environment [128]. During the pulsing operation, the surface charge state and reactant adsorption behavior on the electrode are temporally tuned, which facilitates the accumulation of key intermediates and the regeneration of active species [129].

**FIGURE 9:**
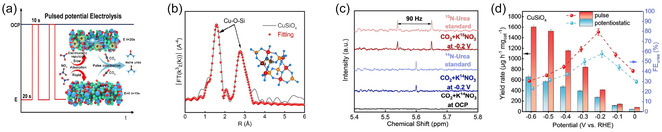
(a) Schematic illustration of the pulsed potential electrolysis strategy. (b) Cu K‐edge EXAFS spectra and corresponding fitting results for the Cu—O—Si catalyst. (c) ^1^H NMR spectra from isotope‐labeling experiments using CO_2_ and ^15^NO_3_^–^, confirming urea formation. (d) Comparison of urea yield and Faradaic efficiency under potentiostatic and pulsed electrolysis conditions over the CuSiO_*x*_ catalyst. Adapted with permission from Qiu et al. [131]. Copyright 2025, Wiley‐VCH GmbH.

Qiu et al. employed a pulsed potential electrolysis strategy to dynamically modulate the catalytic surface environment, thereby achieving a significant enhancement in urea synthesis over a CuSiO_*x*_ nanotube catalyst [131]. This approach was designed to address the antagonistic adsorption behavior of the two reactants during the coreduction process. As illustrated in the mechanistic scheme, under conventional constant potential conditions, the catalyst surface becomes negatively charged, which facilitates CO_2_ adsorption but simultaneously hinders NO_3_^–^ adsorption due to electrostatic repulsion, ultimately suppressing C—N coupling (Figure 9a). The pulsed potential protocol effectively overcomes this limitation by periodically alternating between cathodic and anodic potentials. During the anodic interval, the electrostatic repulsion at the catalyst surface is alleviated, enabling rapid adsorption of NO_3_^–^. Subsequently, the cathodic pulse promotes the coadsorption of CO_2_ and NO_3_^–^ species, thereby accelerating the formation of C—N bonds. Structural characterization by FT‐EXAFS confirmed that CuSiO_*x*_ contains abundant Cu—O—Si interfacial sites, which serve as the active centers for the coupling reaction (Figure 9b). In addition, the accuracy of the synthesized product was validated by ^1^H NMR analysis (Figure 9c). As shown in the performance comparison, the pulsed potential operation (red bars) consistently outperformed the conventional constant potential mode (blue bars) across the entire potential range. Notably, at –0.2 V (vs. RHE), the CuSiO_*x*_ catalyst achieved a high urea yield rate of 1.606 mg h^−1^ mg_cat_^−1^ and an outstanding FE of 79.0% (Figure 9d). These results highlight that the pulsed electrolysis strategy provides an effective means of controlling reactant adsorption dynamics on the catalyst surface, thereby maximizing C—N coupling efficiency [130].

In summary, pulsed electrolysis provides a dynamic approach to regulate the interfacial reaction environment by periodically modulating the applied potential. This strategy enables temporal control over surface charge states and reactant adsorption behavior, thereby facilitating the formation and coupling of key intermediates. By overcoming the limitations of static potential conditions, pulsed operation can effectively balance competing reactions and enhance both reaction rate and product selectivity. These findings highlight pulsed electrolysis as a powerful reaction engineering strategy for improving the efficiency and controllability of electrochemical C—N coupling.

## 6 Outlooks

The electrochemical coupling of carbon sources such as CO_2_ and CO with reactive nitrogen species (NO_*x*_, NO_2_^−^, NO_3_^−^) represents a pivotal frontier in sustainable catalysis, holding the potential to bridge carbon capture, nitrogen recycling, and value‐added chemical synthesis within a single circular framework. By directly forming C—N bonds from abundant small molecules, this strategy provides an environmentally benign alternative to conventional thermochemical routes that require high temperatures, elevated pressures, or hazardous reagents. Despite the recent surge of interest, however, electrochemical C—N coupling remains constrained by sluggish reaction kinetics, overlapping reduction pathways, and limited selectivity toward desired organonitrogen products [132]. Meaningful progress will depend not only on the design of more active and selective catalysts but also on establishing an integrated understanding of reaction mechanisms, interfacial charge dynamics, and reactor‐level phenomena [133]. In this section, we highlight the main scientific challenges and propose forward‐looking strategies to guide the next generation of research in this emerging field.

(1) Quantitative detection and mechanistic visualization: The unambiguous identification of C—N coupling products and intermediates remains a fundamental challenge. In situ and operando spectroscopies, including infrared (IR), Raman, UV–vis, and X‐ray absorption spectroscopy (XAS), have enabled the real‐time monitoring of adsorbed intermediates and dynamic active‐site evolution. These techniques have revealed transient species like ^*^COOH, ^*^CO, ^*^NO, and ^*^NH_2_, helping clarify how carbon and nitrogen moieties form C—N bonds. Yet, each analytical technique possesses intrinsic limitations. For example, UV–vis spectroscopy often suffers from spectral overlap and fluorescence background, while Raman and IR may struggle with weak signal intensity in aqueous media. To obtain more reliable mechanistic information, multimodal characterization is essential; combining surface‐enhanced Raman scattering (SERS), synchrotron‐based XAS, and DEMS can provide complementary insights into electronic structure, molecular identity, and reaction kinetics. Moreover, coupling these methods with machine‐learning‐assisted spectral deconvolution could help quantify reaction rates for individual intermediates. Such integrated operando analytics will be indispensable for unraveling the catalytic cycle and identifying the microscopic factors that control C—N bond formation.

(2) Rational control of intermediate adsorption: Selective C—N bond formation critically depends on precise control of ^*^CO_*x*_ and ^*^NH_*x*_ adsorption on the catalyst surface. The delicate balance between adsorption strength and surface mobility determines whether the intermediates undergo coupling, desorption, or competing side reactions. Designing catalysts with dual‐atom or heteroatomic active sites provides a promising path forward. Bimetallic centers such as Cu—Ni or Cu—Mn exhibit cooperative behavior, where one site stabilizes carbon intermediates, and the other activates nitrogen species, thereby reducing the kinetic barrier for ^*^CO–^*^NH_2_ coupling [134, 135]. Electronic‐structure modulation through heterostructuring or vacancy engineering can further optimize the d‐band center, fine‐tuning adsorption energies to favor C—N coupling over CO_2_RR or HER. Environmental factors, including electrolyte composition, pH, and local electric field, also play nontrivial roles. Alkaline electrolytes, for instance, suppress proton activity and mitigate HER, whereas buffers containing nitrate or nitrite facilitate nitrogen activation. Future studies should integrate theoretical screening with microkinetic modeling to establish quantitative correlations between electronic structure, adsorption energetics, and overall selectivity. Through such efforts, rational control of intermediate binding could evolve from empirical tuning to predictive design.

(3) Suppression of competing pathways: Electrochemical C—N coupling inherently competes with several side reactions, notably the hydrogen evolution reaction (HER), CO_2_ reduction to hydrocarbons, and independent NO_*x*_ reduction pathways [136]. These parallel processes not only reduce carbon and nitrogen utilization efficiency but also complicate mechanistic interpretation. To minimize such competition, spatial and electronic separation of active sites has proven effective. Dual‐functional catalysts that segregate CO_2_ and NO_*x*_ reduction domains can guide electron flow preferentially toward coupling intermediates. Similarly, surface charge engineering such as doping, interface polarization, or external electric fields can bias the local potential landscape to suppress undesired reactions. Adjusting the electrolyte identity and pH remains a powerful yet underutilized lever for steering reaction selectivity. Looking forward, integrating computational potential‐dependent energy maps with experimental operando spectroscopy is expected to enable dynamic control of the reaction environment. Ultimately, achieving near‐exclusive C—N coupling requires not only active catalysts but also smart reaction orchestration that harmonizes charge transfer, mass transport, and interfacial energetics.

(4) Reactor and system level engineering: While catalyst innovation remains the core of C—N coupling research, advancements at the reactor and system level are equally important for translating laboratory discoveries into scalable technologies [64]. Conventional H‐cell configurations, though valuable for mechanistic studies, are limited by mass‐transfer constraints and inefficient product separation [137]. In contrast, flow‐cell and gas‐diffusion electrode architectures enable continuous reactant supply and overcome mass transport limitations, achieving industrially relevant current densities in the hundreds of mA cm^−2^ or even higher, thus highlighting their strong potential for practical implementation [138, 139]. Tandem reactor designs, which couple sequential electrochemical reduction in sequential chambers, can offer additional flexibility by allowing independent optimization of each half‐reaction [140]. Moreover, integrating these systems with renewable electricity sources, such as photovoltaic or wind power, can further improve the overall environmental sustainability. Realizing these system‐level optimizations requires close collaboration among electrochemists, materials scientists, and process engineers to establish reliable performance metrics, including Faradaic efficiency, turnover frequency, and energy conversion efficiency under practical operation conditions.

(5) Technoeconomic and environmental assessment: Beyond scientific discovery, the future of electrochemical C—N coupling will depend on both its technoeconomic feasibility and environmental footprint. Comprehensive cradle‐to‐grave analyses are urgently needed to evaluate capital costs, catalyst lifetime, energy consumption, and product recovery. Benchmarking these parameters against existing ammonia‐ or urea‐based processes will clarify the competitiveness of electrochemical routes. Equally important is the life‐cycle assessment (LCA) to quantify the net reduction in CO_2_ emissions and nitrate pollutants achieved per unit of organonitrogen product. By establishing transparent metrics and standardized testing protocols, the community can identify key cost drivers and environmental trade‐offs. Moreover, integrating digital twins and process simulations will also accelerate scale‐up by linking laboratory kinetics with industrial reactor design.

A distinctive contribution of this review lies in its integrated perspective that explicitly connects reaction mechanisms, analytical methodologies, and catalyst/reaction‐engineering strategies in electrochemical C—N coupling. In particular, we highlight that the reliable interpretation of C—N coupling pathways is not solely determined by catalyst performance but critically depends on the capabilities and limitations of analytical techniques used to identify intermediates and products. By linking mechanistic insights with experimental observables and catalyst design principles, this review provides a unified framework for interpreting complex reaction networks. Looking forward, further progress in electrochemical C—N coupling will require the coordinated development of these three interdependent dimensions, enabling more selective, efficient, and scalable systems for sustainable organonitrogen synthesis.

In summary, electrochemical C—N coupling has developed from an interesting idea into a rapidly developing research area that unites pollution reduction with green chemical synthesis. Continued progress will require integration across multiple scales, from understanding reaction mechanisms at the atomic level to designing reactors and evaluating sustainability across the entire system. New approaches are likely to emerge when catalyst design, operando analytics, and process intensification are optimized together, guided by selectivity, scalability, and circularity. As these efforts come together, electrochemical C—N coupling will not only change the synthesis of organonitrogen compounds but also reshape the broader field of electrochemical manufacturing, contributing to a sustainable chemical future in which carbon and nitrogen are balanced.

## Funding

This work was supported by the National Supercomputing Center, Korea Institute of Science and Technology Information (KSC‐2024‐CRE‐0141), and The Catholic University of Korea (M‐2025‐B0002‐00029).

## Conflicts of Interest

The authors declare no conflicts of interest.

## Data Availability

The data that support the findings of this study are available from the corresponding author upon reasonable request.

## References

[1] L. J. R. Nunes , “The Rising Threat of Atmospheric CO_2_: A Review on the Causes, Impacts, and Mitigation Strategies,” Environments 10, no. 4 (2023):10, 10.3390/environments10040066.

[2] E. Rueda , V. Senatore , T. Zarra , et al., “Life Cycle Assessment and Economic Analysis of Bioplastics Production from Cyanobacteria,” Sustainable Materials and Technologies 35 (2023): e00579, 10.1016/j.susmat.2023.e00579.

[3] V. Rosca , M. Duca , M. T. de Groot , and M. T. Koper , “Nitrogen Cycle Electrocatalysis,” Chemical Reviews 109, no. 6 (2009): 2209–2244, 10.1021/cr8003696. 19438198

[4] P. De Luna , C. Hahn , D. Higgins , et al., “What Would It Take for Renewably Powered Electrosynthesis to Displace Petrochemical Processes?,” Science 364, no. 6438 (2019): eaav3506, 10.1126/science.aav3506. 31023896

[5] Y. Ye , Z. Li , S. Ding , et al., “Synergistic Treatment of Carbon Dioxide and Nitrogen‐Containing Wastewater by Electrochemical C‐N Coupling,” IScience 26, no. 7 (2023): 107009, 10.1016/j.isci.2023.107009. 37534157 PMC10391661

[6] X. Peng , L. Zeng , D. Wang , et al., “Electrochemical C‐N Coupling of CO_2_ and Nitrogenous Small Molecules for the Electrosynthesis of Organonitrogen Compounds,” Chemical Society Reviews 52, no. 6 (2023): 2193–2237, 10.1039/d2cs00381c. 36806286

[7] Y. Li , S. Zheng , H. Liu , et al., “Sequential Co‐Reduction of Nitrate and Carbon Dioxide Enables Selective Urea Electrosynthesis,” Nature Communications 15, no. 1 (2024): 176, 10.1038/s41467-023-44131-z. PMC10761727 38167809

[8] R. J. Detz , C. J. Ferchaud , A. J. Kalkman , et al., “Electrochemical CO_2_ Conversion Technologies: State‐of‐the‐Art and Future Perspectives,” Sustainable Energy & Fuels 7, no. 23 (2023): 5445–5472, 10.1039/d3se00775h.

[9] A. Hassan , M. Afshari , and M. Rahimi , “A Membraneless Electrochemically Mediated Amine Regeneration for Carbon Capture,” Nature Communications 16, no. 1 (2025): 6333, 10.1038/s41467-025-61525-3. PMC12241579 40634332

[10] Y. Ren , C. Yu , X. Tan , et al., “Strategies to Activate Inert Nitrogen Molecules for Efficient Ammonia Electrosynthesis: Current Status, Challenges, and Perspectives,” Energy & Environmental Science 15, no. 7 (2022): 2776–2805, 10.1039/d2ee00358a.

[11] C. Choi , G. H. Gu , J. Noh , H. S. Park , and Y. Jung , “Understanding Potential‐Dependent Competition between Electrocatalytic Dinitrogen and Proton Reduction Reactions,” Nature Communications 12, no. 1 (2021): 4353, 10.1038/s41467-021-24539-1. PMC8285508 34272379

[12] Y. Zhou , C. Ding , and C. Li , “Electrocatalytic Synthesis of Organonitrogen Compounds via C−N Coupling from NO_x_ and Carbon Source,” ChemCatChem 16, no. 23 (2024): e202400941, 10.1002/cctc.202400941.

[13] S. Wu and F. Liu , “Recent Progress in the Electrochemical Formation of C‐N Bonds for Construction of Organic Compounds via the use of NO_x_/NO_x_ ^−^ ,” ChemSusChem 18, no. 4 (2025): e202401751, 10.1002/cssc.202401751. 39375153

[14] Y. Guo , Y. Li , X. Wang , L. Wang , and Z. Wang , “Recent Advances in Electrochemical C–N Coupling for Carbon and Nitrogen Emissions Reduction and Resource Recovery,” Chemical Engineering Journal 499 (2024): 155920, 10.1016/j.cej.2024.155920.

[15] C. Zhao , Y. Jin , J. Yuan , et al., “Tailoring Activation Intermediates of CO_2_ Initiates C‐N Coupling for Highly Selective Urea Electrosynthesis,” Journal of the American Chemical Society 147, no. 10 (2025): 8871–8880, 10.1021/jacs.5c00583. 40035438

[16] H. Zhao , Z. Li , J. Xiang , W. Du , and K. Chu , “Atomically Dispersed Ru on Cu_3_N for Electrocatalytic Reduction of CO_2_ and Nitrite to Urea,” Chemical Engineering Journal 496 (2024): 154256, 10.1016/j.cej.2024.154256.

[17] S. Zhang , J. Geng , Z. Zhao , et al., “High‐Efficiency Electrosynthesis of Urea over Bacterial Cellulose Regulated Pd–Cu Bimetallic Catalyst,” EES Catalysis 1, no. 1 (2023): 45–53, 10.1039/d2ey00038e.

[18] H. Wang , Y. Jiang , S. Li , et al., “Realizing Efficient C‐N Coupling via Electrochemical Co‐Reduction of CO_2_ and NO_3_ ^−^ on AuPd Nanoalloy to Form Urea: Key C‐N Coupling Intermediates,” Applied Catalysis B: Environmental 318 (2022): 121819, 10.1016/j.apcatb.2022.121819.

[19] N. Cao , Y. Quan , A. Guan , et al., “Oxygen Vacancies Enhanced Cooperative Electrocatalytic Reduction of Carbon Dioxide and Nitrite Ions to Urea,” Journal of Colloid and Interface Science 577 (2020): 109–114, 10.1016/j.jcis.2020.05.014. 32473474

[20] Z. Li , P. Zhou , M. Zhou , et al., “Synergistic Electrocatalysis of Crystal Facet and O‐Vacancy for Enhancive Urea Synthesis from Nitrate and CO_2_ ,” Applied Catalysis B: Environmental 338 (2023): 122962, 10.1016/j.apcatb.2023.122962.

[21] X. Wei , Y. Liu , X. Zhu , et al., “Dynamic Reconstitution between Copper Single Atoms and Clusters for Electrocatalytic Urea Synthesis,” Advanced Materials 35, no. 18 (2023): 2300020, 10.1002/adma.202300020. 36744440

[22] L. Kong , D. Jiao , Z. Wang , et al., “Single Metal Atom Anchored on Porous Boron Nitride Nanosheet for Efficient Collaborative Urea Electrosynthesis: A Computational Study,” Chemical Engineering Journal 451, no. 3 (2023): 138885, 10.1016/j.cej.2022.138885.

[23] S. Zhou , Y. Shi , Y. Dai , et al., “Continuous‐Flow Electrosynthesis of Urea and Oxalic Acid by CO_2_‐Nitrate Reduction and Glycerol Oxidation,” Chinese Journal of Catalysis 63 (2024): 270–281, 10.1016/s1872-2067(24)60085-9.

[24] M. Li , Q. Shi , Z. Li , et al., “Photoelectrocatalytic Synthesis of Urea from Carbon Dioxide and Nitrate over a Cu2O Photocathode,” Angewandte Chemie International Edition 136, no. 33 (2024): e202406515, 10.1002/ange.202406515. 38803131

[25] X. Zhang , Y. Lyu , C. Chen , et al., “Enhanced Charge‐Carrier Dynamics and Efficient Solar‐to‐Urea Conversion on Si‐Based Photocathodes,“Proceedings of the National Academy of Sciences of the United States of America 121, no. 8 (2024): e2311326121, 10.1073/pnas.2311326121. 38349884 PMC10895350

[26] C. Han and K. Wang , “Recent Advances in Photoelectrochemical Synthesis of Nitrogen‐Containing Solar Fuels and Chemicals,” Energy & Fuels 39, no. 34 (2025): 16065–16077, 10.1021/acs.energyfuels.5c02821.

[27] P. Ramadhany , T. Trần‐Phú , J. A. Yuwono , et al., “Triggering C‐N Coupling on Metal Oxide Nanocomposite for the Electrochemical Reduction of CO_2_ and NO* _x_ * ⁻ to Formamide,” Advanced Energy Materials 14, no. 32 (2024): 2401786, 10.1002/aenm.202401786.

[28] Y. Luo , K. Xie , P. Ou , et al., “Selective Electrochemical Synthesis of Urea from Nitrate and CO_2_ via Relay Catalysis on Hybrid Catalysts,” Nature Catalysis 6, no. 10 (2023): 939–948, 10.1038/s41929-023-01020-4.

[29] K. Yu , H. Wang , W. Yu , et al., “Resource Utilization of Carbon Dioxide and Nitrate to Produce Value‐Added Organonitrogen Compounds through an Electrochemical Approach,” Applied Catalysis B: Environmental 341 (2024): 123292, 10.1016/j.apcatb.2023.123292.

[30] Y. Wang , D. Chen , C. Chen , and S. Wang , “Electrocatalytic Urea Synthesis via C‐N Coupling from CO_2_ and Nitrogenous Species,” Accounts of Chemical Research 57, no. 2 (2024): 247–256, 10.1021/acs.accounts.3c00633. 38129325

[31] M. Zhou , Y. Zhang , H. Li , et al., “Tailoring O‐Monodentate Adsorption of CO_2_ Initiates C‐N Coupling for Efficient Urea Electrosynthesis with Ultrahigh Carbon Atom Economy,” Angewandte Chemie International Edition 64, no. 2 (2025): e202414392, 10.1002/anie.202414392. 39180230

[32] T. Cheng , H. Xiao , and W. A. Goddard III , “Reaction Mechanisms for the Electrochemical Reduction of CO_2_ to CO and Formate on the Cu(100) Surface at 298 K from Quantum Mechanics Free Energy Calculations with Explicit Water,” Journal of the American Chemical Society 138, no. 42 (2016): 13802–13805, 10.1021/jacs.6b08534. 27726392

[33] G. T. K. Kalhara Gunasooriya and M. Saeys , “CO Adsorption Site Preference on Platinum: Charge Is the Essence,” ACS Catalysis 8, no. 5 (2018): 3770–3774, 10.1021/acscatal.8b00214.

[34] X. Chang , H. Xiong , Q. Lu , and B. Xu , “Mechanistic Implications of Low CO Coverage on Cu in the Electrochemical CO and CO_2_ Reduction Reactions,” JACS Au 3, no. 11 (2023): 2948–2963, 10.1021/jacsau.3c00494. 38034971 PMC10685414

[35] H. Liu , J. Park , Y. Chen , et al., “Electrocatalytic Nitrate Reduction on Oxide‐Derived Silver with Tunable Selectivity to Nitrite and Ammonia,” ACS Catalysis 11, no. 14 (2021): 8431–8442, 10.1021/acscatal.1c01525.

[36] M. Karamad , T. J. Goncalves , S. Jimenez‐Villegas , I. D. Gates , and S. Siahrostami , “Why Copper Catalyzes Electrochemical Reduction of Nitrate to Ammonia,” Faraday Discussions 243, no. 0 (2023): 502–519, 10.1039/d2fd00145d. 37051713

[37] J. Long , D. Luan , X. Fu , H. Li , and J. Xiao , “Theoretical Design of the Electrocatalytic Urea Synthesis from Carbon Dioxide and Nitric Oxides,” ACS Catalysis 14, no. 19 (2024): 14678–14687, 10.1021/acscatal.4c03785.

[38] L. Xu , Z. Yang , C. Zhang , and C. Chen , “Recent Progress in Electrochemical C‐N Coupling: Metal Catalyst Strategies and Applications,” Chemical Communications 60, no. 78 (2024): 10822–10837, 10.1039/d4cc03256j. 39233628

[39] H. Cai , J. Ding , T. Hou , et al., “Recent Progress in Electrochemical Synthesis of Urea through C‐N Coupling Reactions,” Chemical Synthesis 4, no. 4 (2024): 15, 10.20517/cs.2024.15.

[40] H. Ooka , J. Huang , and K. S. Exner , “The Sabatier Principle in Electrocatalysis: Basics, Limitations, and Extensions,” Frontiers in Energy Research 9 (2021): 654460, 10.3389/fenrg.2021.654460.

[41] D. Anastasiadou and M. Costa Figueiredo , “Electrocatalytic Pathways to the Formation of C–N Bonds,” ACS Catalysis 14, no. 7 (2024): 5088–5097, 10.1021/acscatal.3c04912.

[42] X. Zhang , X. Zhu , S. Bo , et al., “Identifying and Tailoring C‐N Coupling Site for Efficient Urea Synthesis over Diatomic Fe‐Ni Catalyst,” Nature Communications 13, no. 1 (2022): 5337, 10.1038/s41467-022-33066-6. PMC9464195 36088335

[43] Y. Zhou , B. Yang , Z. Huang , et al., “Cu‐Ni Alloy Nanocrystals with Heterogenous Active Sites for Efficient Urea Synthesis,” Applied Catalysis B: Environmental 343 (2024): 123577, 10.1016/j.apcatb.2023.123577.

[44] Z. Dai , Y. Chen , H. Zhang , et al., “Surface Engineering on Bulk Cu(2)O for Efficient Electrosynthesis of Urea,” Nature Communications 16, no. 1 (2025): 3271, 10.1038/s41467-025-57708-7. PMC11972395 40188148

[45] J. Lan , Z. Wei , Y. R. Lu , et al., “Efficient Electrosynthesis of Formamide from Carbon Monoxide and Nitrite on a Ru‐Dispersed Cu Nanocluster Catalyst,” Nature Communications 14, no. 1 (2023): 2870, 10.1038/s41467-023-38603-5. PMC10198976 37208321

[46] X. Zhu , X. Zhou , Y. Jing , and Y. Li , “Electrochemical Synthesis of Urea on MBenes,” Nature Communications 12, no. 1 (2021): 4080, 10.1038/s41467-021-24400-5. PMC8253759 34215749

[47] B. Xu , D. Li , Q. Zhao , et al., “Electrochemical Reduction of Nitrate to Ammonia Using Non‐Precious Metal‐Based Catalysts,” Coordination Chemistry Reviews 502 (2024): 215609, 10.1016/j.ccr.2023.215609.

[48] Y. Zhao , Y. Ding , W. Li , et al., “Efficient Urea Electrosynthesis from Carbon Dioxide and Nitrate via Alternating Cu‐W Bimetallic C‐N Coupling Sites,” Nature Communications 14, no. 1 (2023): 4491, 10.1038/s41467-023-40273-2. PMC10372083 37495582

[49] Y. Xu , G. D. Foley , L. On , V. S. Thoi , and F. Che , “Multi‐Scale Modeling Guided Electrochemical C–N Coupling for Urea Production in Metal‐Organic Frameworks,” Journal of Catalysis 453 (2026): 116523, 10.1016/j.jcat.2025.116523.

[50] S. Kuang , T. Xiao , H. Chi , et al., “Acetamide Electrosynthesis from CO_2_ and Nitrite in Water,” Angewandte Chemie International Edition 63, no. 9 (2024): 202316772, 10.1002/anie.202316772. 38204294

[51] Y. Wu , Z. Jiang , Z. Lin , Y. Liang , and H. Wang , “Direct Electrosynthesis of Methylamine from Carbon Dioxide and Nitrate,” Nature Sustainability 4, no. 8 (2021): 725–730, 10.1038/s41893-021-00705-7.

[52] Y. Fan , T. Liu , Y. Yan , et al., “Electrochemical Synthesis of Formamide by C–N Coupling with Amine and CO_2_ with a High Faradaic Efficiency of 37.5%,” Chem 10, no. 8 (2024): 2437–2449, 10.1016/j.chempr.2024.03.024.

[53] Y. Zhou , R. Duan , L. Liu , C. Ding , and C. Li , “Electrocatalytic Synthesis of Methylamine from Nitrate and Carbon Dioxide on a Heterometallic Polyphthalocyanine,” Chemical Science 16, no. 37 (2025): 17148–17155, 10.1039/d5sc04641f. 40896320 PMC12395663

[54] G. ‐L. Yang , C. ‐T. Hsieh , Y. ‐S. Ho , et al., “Gaseous CO_2_ Coupling with N‐Containing Intermediates for Key C–N Bond Formation during Urea Production from Coelectrolysis over Cu,” ACS Catalysis 12, no. 18 (2022): 11494–11504, 10.1021/acscatal.2c02346.

[55] J. Tao , C. Wang , Y. Zhu , and J. Shen , “Nitrogen‐Doped Carbon‐Armored Copper–nickel Alloy Electrocatalytic C–N Coupling for Urea Synthesis,” AIChE Journal 71, no. 11 (2025): e70048, 10.1002/aic.70048.

[56] H. Han , J. Y. Kim , S. H. Roh , S. Baik , and J. K. Kim , “Tailoring Atomic Metal Species for Electrochemical‐Driven Urea Synthesis Coupled with CO_2_ and Nitrogen Sources in Aqueous Media,” Korean Journal of Chemical Engineering 42, no. 11 (2025): 2415–2437, 10.1007/s11814-024-00343-7.

[57] M. Kumar , B. Meena , P. Subramanyam , D. Suryakala , and C. Subrahmanyam , “Recent Trends in Photoelectrochemical Water Splitting: The Role of Cocatalysts,” NPG Asia Materials 14, no. 1 (2022): 88, 10.1038/s41427-022-00436-x.

[58] S. Ha , D. ‐K. Oh , D. Choi , et al., “Carbon‐Based Catalysts for Photoelectrochemical Water Splitting: Advancing Carbon–neutral Hydrogen Production,” Carbon Neutrality 4, no. 1 (2025): 38, 10.1007/s43979-025-00150-x.

[59] J. Zheng , S. Xu , J. Sun , et al., “Boosting Efficient C‐N Bonding toward Photoelectrocatalytic Urea Synthesis from CO_2_ and Nitrate via Close Cu/Ti Bimetallic Sites,” Applied Catalysis B: Environmental 338 (2023):123056, 10.1016/j.apcatb.2023.123056.

[60] Y. Wan , M. Zheng , W. Yan , J. Zhang , and R. Lv , “Fundamentals and Rational Design of Heterogeneous C‐N Coupling Electrocatalysts for Urea Synthesis at Ambient Conditions,” Advanced Energy Materials 14, no. 28 (2024): 202303588, 10.1002/aenm.202303588.

[61] J. Mukherjee , S. Paul , A. Adalder , et al., “Understanding the Site‐Selective Electrocatalytic Co‐Reduction Mechanism for Green Urea Synthesis Using Copper Phthalocyanine Nanotubes,” Advanced Functional Materials 32, no. 31 (2022): 2200882, 10.1002/adfm.202200882.

[62] Y. Huang , Y. Wang , Y. Liu , et al., “Unveiling the Quantification Minefield in Electrocatalytic Urea Synthesis,” Chemical Engineering Journal 453, no. 1 (2023): 139836, 10.1016/j.cej.2022.139836.

[63] M. Jouny , J. J. Lv , T. Cheng , et al., “Formation of Carbon‐Nitrogen Bonds in Carbon Monoxide Electrolysis,” Nature Chemistry 11, no. 9 (2019): 846–851, 10.1038/s41557-019-0312-z. 31444485

[64] C. Yang , Z. Li , J. Xu , Y. Jiang , and W. Zhu , “Electrocatalytic C‐N Coupling for Urea Synthesis: A Critical Review,” Green Chemistry 26, no. 9 (2024): 4908–4933, 10.1039/d3gc04920e.

[65] W. Shen , Y. Ye , Q. Xia , and P. Xi , “Progress in In Situ Characterization of Electrocatalysis,” EES Catalysis 3, no. 1 (2025): 10–31, 10.1039/d4ey00168k.

[66] D. Lyu , J. Xu , and Z. Wang , “Toward Reliable and Accessible Ammonia Quantification in the Electrocatalytic Reduction of Nitrogen,” Frontiers in Chemistry 11, no. 7 (2023): 1505–1518, 10.3389/fchem.2023.1231886.

[67] G. Li , Z. ‐C. An , J. Yang , et al., “Revealing Surface Fine Structure on PtAu Catalysts by an In Situ ATR‐SEIRAS CO‐Probe Method,” Journal of Materials Chemistry A 11, no. 26 (2023): 14043–14051, 10.1039/d3ta01668d.

[68] Y. Shang , D. Chen , C. Chen , and S. Wang , “Surface‐Functionalized Two‐Dimensional Materials towards Electrocatalytic C−N Coupling Reaction for Urea,” Catalysis 1, no. 1 (2025): 9. 10.1007/s44422-025-00009-3.

[69] M. C. G. Pella , A. R. Simao , P. Valderrama , and A. F. Rubira , “A Conventional and Chemometric Analytical Approach to Solving Urea Determination with Accuracy and Precision,” Analytical Methods 15, no. 16 (2023): 2016–2029, 10.1039/d3ay00249g. 37060118

[70] S. Chen , S. Lin , L. X. Ding , and H. Wang , “Modified Diacetylmonoxime‐Thiosemicarbazide Detection Protocol for Accurate Quantification of Urea,” Small Methods 7, no. 9 (2023): e2300003, 10.1002/smtd.202300003. 37330664

[71] Y. Chang , T. E. Park , S. W. Lee , and E. H. Lee , “Colorimetric Detection of Urease‐Producing Microbes Using an Ammonia‐Responsive Flexible Film Sensor,” Biosensors 12, no. 10 (2022): 886, 10.3390/bios12100886. 36291023 PMC9599750

[72] T. Yuan and O. Voznyy , “Guidelines for Reliable Urea Detection in Electrocatalysis,” Cell Reports Physical Science 4, no. 8 (2023): 101521, 10.1016/j.xcrp.2023.101521.

[73] W. P. Utomo , H. Wu , and Y. H. Ng , “Quantification Methodology of Ammonia Produced from Electrocatalytic and Photocatalytic Nitrogen/Nitrate Reduction,” Energies 16, no. 1 (2023): 27, 10.3390/en16010027.

[74] A. J. Martín , F. L. P. Veenstra , J. Lüthi , R. Verel , and J. Pérez‐Ramírez , “Toward Reliable and Accessible Ammonia Quantification in the Electrocatalytic Reduction of Nitrogen,” Chem Catalysis 1, no. 7 (2021): 1505–1518, 10.1016/j.checat.2021.10.002.

[75] T. Beyazay , W. F. Martin , and H. Tuysuz , “Direct Synthesis of Formamide from CO_2_ and H_2_O with Nickel‐Iron Nitride Heterostructures under Mild Hydrothermal Conditions,” Journal of the American Chemical Society 145, no. 36 (2023): 19768–19779, 10.1021/jacs.3c05412. 37642297 PMC7615090

[76] D. A. Jackson and S. A. Mabury , “Polyfluorinated Amides as a Historical PFCA Source by Electrochemical Fluorination of Alkyl Sulfonyl Fluorides,” Environmental Science & Technology 47, no. 1 (2013): 382–389, 10.1021/es303152m. 23205559

[77] H. Hecht , W. Y. Rojas , Z. Ahmad , et al., “Quantum Chemistry‐Based Prediction of Electron Ionization Mass Spectra for Environmental Chemicals,” Analytical Chemistry 96, no. 33 (2024): 13652–13662, 10.1021/acs.analchem.4c02589. 39110763 PMC11339729

[78] Z. Mo , J. Mu , and B. Liu , “Advances in Electrocatalytic Urea Synthesis: From Fundamentals to Applications,” Advanced Powder Materials 3, no. 6 (2024): 100245, 10.1016/j.apmate.2024.100245.

[79] S. Paul , S. Sarkar , A. Adalder , A. Banerjee , and U. K. Ghorai , “Dual Metal Site‐Mediated Efficient C‐N Coupling toward Electrochemical Urea Synthesis,” Journal of Materials Chemistry A 11, no. 25 (2023): 13249–13254, 10.1039/d3ta01011b.

[80] S. Fu , K. Chu , M. Guo , et al., “Ultrasonic‐Assisted Hydrothermal Synthesis of RhCu Alloy Nanospheres for Electrocatalytic Urea Production,” Chemical Communications 59, no. 29 (2023): 4344–4347, 10.1039/d3cc00102d. 36946147

[81] E. L. Clark , M. R. Singh , Y. Kwon , and A. T. Bell , “Differential Electrochemical Mass Spectrometer Cell Design for Online Quantification of Products Produced during Electrochemical Reduction of CO_2_ ,” Analytical Chemistry 87, no. 15 (2015): 8013–8020, 10.1021/acs.analchem.5b02080. 26153829

[82] K. Zhao , X. Jiang , X. Wu , et al., “Recent Development and Applications of Differential Electrochemical Mass Spectrometry in Emerging Energy Conversion and Storage Solutions,” Chemical Society Reviews 53, no. 13 (2024): 6917–6959, 10.1039/d3cs00840a. 38836324

[83] J. Leverett , T. Tran‐Phu , J. A. Yuwono , et al., “Tuning the Coordination Structure of Cu—N—C Single Atom Catalysts for Simultaneous Electrochemical Reduction of CO_2_ and NO_3_– to Urea,” Advanced Energy Materials 12, no. 32 (2022): 2201500, 10.1002/aenm.202201500.

[84] S. Liu , S. Yin , Z. Wang , et al., “AuCu Nanofibers for Electrosynthesis of Urea from Carbon Dioxide and Nitrite,” Cell Reports Physical Science 3, no. 5 (2022): 100869, 10.1016/j.xcrp.2022.100869.

[85] Y. Huang , R. Yang , C. Wang , et al., “Direct Electrosynthesis of Urea from Carbon Dioxide and Nitric Oxide,” ACS Energy Letters 7, no. 1 (2022): 284–291, 10.1021/acsenergylett.1c02471.

[86] Z. Xi , H. Hu , Q. Chen , et al., “2D Catalysts for Electrocatalytic Nitrate Reduction and C–N Coupling Reactions,” Advanced Functional Materials 35, no. 33 (2025): e202425611, 10.1002/adfm.202425611.

[87] B. H. Ko , B. Hasa , H. Shin , et al., “The Impact of Nitrogen Oxides on Electrochemical Carbon Dioxide Reduction,” Nature Communications 11, no. 1 (2020): 5856, 10.1038/s41467-020-19731-8. PMC7672067 33203886

[88] H. ‐Q. Yin , Z. ‐S. Sun , Q. ‐P. Zhao , et al., “Electrochemical Urea Synthesis by Co‐Reduction of CO_2_ and Nitrate with FeII‐FeIIIOOH@BiVO_4_ Heterostructures,” Journal of Energy Chemistry 84 (2023): 385–393, 10.1016/j.jechem.2023.05.032.

[89] J. Qin , N. Liu , L. Chen , et al., “Selective Electrochemical Urea Synthesis from Nitrate and CO_2_ Using In Situ Ru Anchoring onto a Three‐Dimensional Copper Electrode,” ACS Sustainable Chemistry & Engineering 10, no. 48 (2022): 15869–15875, 10.1021/acssuschemeng.2c05110.

[90] N. Li , H. Gao , Z. Liu , et al., “Metalphthalocyanine Frameworks Grown on TiO_2_ Nanotubes for Synergistically and Efficiently Electrocatalyzing Urea Production from CO_2_ and Nitrate,” Science China Chemistry 66, no. 5 (2023): 1417–1424, 10.1007/s11426-023-1524-4.

[91] Y. Mao , Y. Jiang , H. Liu , et al., “Ambient Electrocatalytic Synthesis of Urea by Co‐Reduction of NO_3_ ^−^ and CO_2_ over Graphene‐Supported In_2_O_3_ ,” Chinese Chemical Letters 35, no. 3 (2024): 108540, 10.1016/j.cclet.2023.108540.

[92] M. Cheng , S. Wang , Z. Dai , et al., “Rectifying Heterointerface Facilitated C‐N Coupling Dynamics Enables Efficient Urea Electrosynthesis under Ultralow Potentials,” Angewandte Chemie International Edition 64, no. 1 (2025): e202413534, 10.1002/anie.202413534. 39319367

[93] R. Wang , Y. Liu , Y. Kong , Q. Chen , and S. Zhao , “Boosting Synergistic Catalysis C–N Coupling via Stabilizing Close Zn/Ti Bimetallic Sites for Electrocatalytic Urea Synthesis from CO_2_ and Nitrite,” ACS Catalysis 15, no. 3 (2025): 2703–2714, 10.1021/acscatal.4c08000.

[94] Y. Wang , S. Xia , K. Chen , et al., “Atomic‐Scale Tailoring C‐N Coupling Sites for Efficient Acetamide Electrosynthesis over Cu‐Anchored Boron Nitride Nanosheets,” ACS Nano 18, no. 50 (2024): 34403–34414, 10.1021/acsnano.4c14039. 39630435

[95] H. Jing , J. Long , H. Li , X. Fu , and J. Xiao , “Computational Insights on Electrocatalytic Synthesis of Methylamine from Nitrate and Carbon Dioxide,” ACS Catalysis 13, no. 15 (2023): 9925–9935, 10.1021/acscatal.3c01592.

[96] N. Meng , X. Ma , C. Wang , et al., “Oxide‐Derived Core‐Shell Cu@Zn Nanowires for Urea Electrosynthesis from Carbon Dioxide and Nitrate in Water,” ACS Nano 16, no. 6 (2022): 9095–9104, 10.1021/acsnano.2c01177. 35657689

[97] Y. Yu , Y. Sun , J. Han , et al., “Achieving Efficient Urea Electrosynthesis through Improving the Coverage of a Crucial Intermediate across a Broad Range of Nitrate Concentrations,” Energy & Environmental Science 17, no. 14 (2024): 5183–5190, 10.1039/d4ee01878h.

[98] M. Xu , F. Wu , Y. Zhang , et al., “Kinetically Matched C‐N Coupling toward Efficient Urea Electrosynthesis Enabled on Copper Single‐Atom Alloy,” Nature Communications 14, no. 1 (2023): 6994, 10.1038/s41467-023-42794-2. PMC10620222 37914723

[99] K. Chen , D. Ma , Y. Zhang , et al., “Urea Electrosynthesis from Nitrate and CO_2_ on Diatomic Alloys,” Advanced Materials 36, no. 30 (2024): 2402160, 10.1002/adma.202402160. 38876146

[100] P. Zhan , J. Zhuang , S. Yang , et al., “Efficient Electrosynthesis of Urea over Single‐Atom Alloy with Electronic Metal Support Interaction,” Angewandte Chemie International Edition 63, no. 33 (2024): e202409019, 10.1002/anie.202409019. 38785222

[101] C. Lv , C. Lee , L. Zhong , et al., “A Defect Engineered Electrocatalyst that Promotes High‐Efficiency Urea Synthesis under Ambient Conditions,” ACS Nano 16, no. 5 (2022): 8213–8222, 10.1021/acsnano.2c01956. 35362943

[102] X. Wei , X. Wen , Y. Liu , et al., “Oxygen Vacancy‐Mediated Selective C‐N Coupling toward Electrocatalytic Urea Synthesis,” Journal of the American Chemical Society 144, no. 26 (2022): 11530–11535, 10.1021/jacs.2c03452. 35748598

[103] Y. Mao , F. Ren , Q. Gou , et al., “Enhanced Performance of Oxygen Vacancy‐Rich In‐TiO_2_ Materials for Electrocatalytic Urea Synthesis via a Relay Catalysis Strategy,” Chemical Engineering Journal 485 (2024): 150052, 10.1016/j.cej.2024.150052.

[104] P. Li , Q. Zhu , J. Liu , et al., “Efficient C‐N Coupling for Urea Electrosynthesis on Defective Co_3_O_4_ with Dual‐Functional Sites,” Chemical Science 15, no. 9 (2024): 3233–3239, 10.1039/d3sc06579k. 38425518 PMC10901497

[105] N. Meng , Y. Huang , Y. Liu , Y. Yu , and B. Zhang , “Electrosynthesis of Urea from Nitrite and CO_2_ over Oxygen Vacancy‐Rich ZnO Porous Nanosheets,” Cell Reports Physical Science 2, no. 3 (2021): 100378, 10.1016/j.xcrp.2021.100378.

[106] Z. Li , M. Xu , J. Wang , et al., “Boosting up Electrosynthesis of Urea with Nitrate and Carbon Dioxide via Synergistic Effect of Metallic Iron Cluster and Single‐Atom,” Small 20, no. 38 (2024): e2400036, 10.1002/smll.202400036. 38747043

[107] X. Tu , X. Zhu , S. Bo , et al., “A Universal Approach for Sustainable Urea Synthesis via Intermediate Assembly at the Electrode/Electrolyte Interface,” Angewandte Chemie International Edition 136, no. 3 (2024): 202317087, 10.1002/ange.202317087. 38055225

[108] M. Yuan , J. Chen , Y. Bai , et al., “Electrochemical C‐N Coupling with Perovskite Hybrids toward Efficient Urea Synthesis,” Chemical Science 12, no. 17 (2021): 6048–6058, 10.1039/d1sc01467f. 33996000 PMC8098680

[109] Z. Wang , R. Zhang , Y. Wang , et al., “Urea Synthesis via Electrocatalytic C‐N Coupling of CO_2_ and Nitrate on Oxygen‐Vacancy‐Rich Co_3_O_4_‐CuO Heterostructure Nanowires,” Chemical Communications 61, no. 52 (2025): 9496–9499, 10.1039/d5cc01629k. 40454581

[110] W. X. Liang , H. Han , C. H. Zhen , et al., “A Review on Solid‐Solid Heterogeneous Interfacial Interactions in Electrocatalytic Conversion of Inorganic Molecules,” Chemical Communications 60, no. 100 (2024): 14924–14934, 10.1039/d4cc05433d. 39584583

[111] S. Lalwani , M. AlNahyan , A. Al Zaabi , F. AlMarzooqi , and A. Qurashi , “Advances in Interfacial Engineering and Their Role in Heterostructure Formation for HER Applications in Wider pH,” ACS Applied Energy Materials 5, no. 12 (2022): 14571–14592, 10.1021/acsaem.2c02102.

[112] Q. Shen , W. Chen , Z. Wei , et al., “Interfacial Electron Transfer Induced Dual‐Site Synergetic Effects Boosting Formamide Synthesis on CuO/In_2_O_3_ Hollow Cubes,” Applied Catalysis B: Environment and Energy 378 (2025): 125581, 10.1016/j.apcatb.2025.125581.

[113] J. Bai , X. Cai , X. Liu , N. Singh , and L. Yao , “Recent Advances in Microenvironment Engineering for Selective Electrochemical C‐N Coupling,” ChemSusChem 18, no. 20 (2025): e2501366, 10.1002/cssc.202501366. 40891228

[114] X. Yuan , X. Yin , Y. Zhang , and Y. Chen , “High‐Entropy Alloys and Their Derived Compounds as Electrocatalysts: Understanding, Preparation and Application,” Materials 18, no. 17 (2025): 4021, 10.3390/ma18174021. 40942447 PMC12429081

[115] L. Liu , H. Akhoundzadeh , M. Li , and H. Huang , “Alloy Catalysts for Electrocatalytic CO_2_ Reduction,” Small Methods 7, no. 9 (2023): 202300482, 10.1002/smtd.202300482. 37256287

[116] S. E. Cooney , S. G. Duggan , M. R. A. Walls , et al., “Engineering Mechanisms of Proton‐Coupled Electron Transfer to a Titanium‐Substituted Polyoxovanadate‐Alkoxide,” Chemical Science 16, no. 6 (2025): 2886–2897, 10.1039/d4sc06468b. 39822902 PMC11733765

[117] Z. Wu , Y. Zhao , W. Jin , et al., “Recent Progress of Vacancy Engineering for Electrochemical Energy Conversion Related Applications,” Advanced Functional Materials 31, no. 9 (2021): 2009070, 10.1002/adfm.202009070.

[118] H. Hu , J. Wang , P. Tao , et al., “Stability of Single‐Atom Catalysts for Electrocatalysis,” Journal of Materials Chemistry A 10, no. 11 (2022): 5835–5849, 10.1039/d1ta08582d.

[119] Y. Huang , Y. Yu , Y. Yu , and B. Zhang , “Oxygen Vacancy Engineering in Photocatalysis,” Solar RRL 4, no. 8 (2020): 2000037, 10.1002/solr.202000037.

[120] G. Chen , C. He , G. Yan , et al., “Mastering Vacancy Engineering for Electrocatalysis: Insights into Classification, Synthesis, and Characterization,” Nano Materials Science 7 (2025): 172, 10.1016/j.nanoms.2025.05.005.

[121] Z. Wang , M. Chen , G. Lu , et al., “Single‐Atom Catalysts toward Electrocatalytic Urea Synthesis via C‐N Coupling Reactions,” Chemical Communications 61, no. 72 (2025): 13601–13615, 10.1039/d5cc03239c. 40827835

[122] L. Zhang , X. Yang , J. Lin , et al., “On the Coordination Environment of Single‐Atom Catalysts,” Accounts of Chemical Research 58, no. 12 (2025): 1878–1892, 10.1021/acs.accounts.5c00140. 40479510

[123] Y. Ren , J. Wang , M. Zhang , et al., “Locally Ordered Single‐Atom Catalysts for Electrocatalysis,” Angewandte Chemie 17, no. 14 (2025): e202315003, 10.1002/cctc.202500579. 37932862

[124] Y. Wang , S. Xia , K. Chen , et al., “Balancing Intermediates Formation on Atomically Pd‐Bridged Cu/Cu_2_O Interfaces for Kinetics‐Matching Electrocatalytic C Horizontal Line N Coupling Reaction,” Angewandte Chemie International Edition 64, no. 22 (2025): e202503011, 10.1002/anie.202503011. 40113558

[125] W. Luo , J. Liu , Y. Hu , and Q. Yan , “Single and Dual‐Atom Catalysts towards Electrosynthesis of Ammonia and Urea: A Review,” Nanoscale 16, no. 44 (2024): 20463–20483, 10.1039/d4nr02387k. 39435616

[126] N. Meng , J. Shao , H. Li , et al., “Electrosynthesis of Formamide from Methanol and Ammonia under Ambient Conditions,” Nature Communications 13, no. 1 (2022): 5452, 10.1038/s41467-022-33232-w. PMC9481544 36114196

[127] Z. Liu , G. Ma , J. Li , et al., “Directing the Electrochemical C‐N Coupling toward Efficient Amide Synthesis via Ammonia Activation‐Mediated Pathway,” Angewandte Chemie 137, no. 52 (2025): e202518108, 10.1002/ange.202518108. 41137438

[128] C. S. Gerke , M. Klenk , P. Zapol , and V. S. Thoi , “Pulsed‐Ptential Electrolysis Enhances Electrochemical C–N Coupling by Reorienting Interfacial Ions,” ACS Catalysis 13, no. 22 (2023): 14540–14547, 10.1021/acscatal.3c03027.

[129] H. Lee and H. Ren , “Tuning Electrocatalytic Oxygen Reduction Reaction with Dynamic Control of Electrochemical Interfaces,” Journal of the American Chemical Society 146, no. 16 (2024): 13694, 10.1021/jacs.3c13694. 38607685

[130] P. Cheng , Z. Wei , J. Chai , and Y. Zhai , “Pulsed Electrocatalysis: A Potential Strategy for Regulating the Heterogeneous Electrocatalytic Reduction Reaction,” ChemCatChem 17, no. 14 (2025): e202500579, 10.1002/cctc.202500579.

[131] W. Qiu , S. Qin , Y. Li , et al., “Overcoming Electrostatic Interaction via Pulsed Electroreduction for Boosting the Electrocatalytic Urea Synthesis,” Angewandte Chemie International Edition 63, no. 24 (2024): e202402684, 10.1002/anie.202402684. 38597346

[132] S. R. Udayasurian and T. Li , “Recent Research Progress on Building C‐N Bonds via Electrochemical NO_x_ Reduction,” Nanoscale 16, no. 6 (2024): 2805–2819, 10.1039/d3nr06151e. 38240609

[133] Y. Liu , X. Yu , X. Li , et al., “Selective Synthesis of Organonitrogen Compounds via Electrochemical C‐N Coupling on Atomically Dispersed Catalysts,” ACS Nano 18, no. 35 (2024): 23894–23911, 10.1021/acsnano.4c06516. 39160683

[134] Y. Dong , D. Jiao , Z. Jin , et al., “Efficient Urea Synthesis by Coupling Catalyst of Nickel‐Single‐Atom and Copper‐Cluster,” Journal of Materials Science & Technology 236 (2025): 115–121, 10.1016/j.jmst.2024.12.095.

[135] S. Fang , J. Du , X. Wu , and Z. Wu , “Co‐Electroreduction of Nitrite Wastewater and CO_2_ through C‐N Coupling Enabled by Oxygen Vacancy Mediated Charge Transfer,” Applied Catalysis B: Environment and Energy 384 (2026): 126209, 10.1016/j.apcatb.2025.126209.

[136] B. Hu , R. Lu , W. Wang , et al., “Directing the C‐N Coupling Pathway Enables Efficient Urea Electrosynthesis,” Journal of the American Chemical Society 147, no. 25 (2025): 21764–21777, 10.1021/jacs.5c04483. 40503998

[137] Y. Zhou , C. Feng , C. Sun , et al., “Urea Electrosynthesis from Gaseous Nitrogen Oxides and Carbon Dioxide: A Review,” Materials Today Catalysis 11 (2025): 100128, 10.1016/j.mtcata.2025.100128.

[138] M. Wang , L. Lin , Z. Zheng , et al., “Hydrophobized Electrospun Nanofibers of Hierarchical Porosity as the Integral Gas Diffusion Electrode for Full‐pH CO_2_ Electroreduction in Membrane Electrode Assemblies,” Energy & Environmental Science 16, no. 10 (2023): 4423–4431, 10.1039/d3ee01866k.

[139] D. Yureka Imali , E. C. J. Perera , M. N. Kaumal , and D. P. Dissanayake , “High‐Performance Gas Diffusion Electrodes for Next‐Generation CO_2_ Conversion Technologies,” RSC Advances 16, no. 1 (2025): 883–915, 10.1039/d5ra06681f. 41488514 PMC12758373

[140] M. Wang , W. Fang , D. Zhu , et al., “Tandem Design on Electrocatalysts and Reactors for Electrochemical CO_2_ Reduction,” Chinese Journal of Catalysis 69 (2025): 1–16, 10.1016/s1872-2067(24)60209-3.

[141] Y. Hu, D. Wang, B. Hu, et al., “Ultra‐precise ruler for ammonia nitrogen quantification in electrochemical synthesis experiments,” *Analytical Methods* 17, no. 7 (2025): 1493–1502, 10.1039/d4ay02288b 39882591

